# One thousand plant transcriptomes and the phylogenomics of green plants

**DOI:** 10.1038/s41586-019-1693-2

**Published:** 2019-10-23

**Authors:** One Thousand Plant Transcriptomes Initiative, James H. Leebens-Mack, Michael S. Barker, Eric J. Carpenter, Michael K. Deyholos, Matthew A. Gitzendanner, Sean W. Graham, Ivo Grosse, Zheng Li, Michael Melkonian, Siavash Mirarab, Martin Porsch, Marcel Quint, Stefan A. Rensing, Douglas E. Soltis, Pamela S. Soltis, Dennis W. Stevenson, Kristian K. Ullrich, Norman J. Wickett, Lisa DeGironimo, Patrick P. Edger, Ingrid E. Jordon-Thaden, Steve Joya, Tao Liu, Barbara Melkonian, Nicholas W. Miles, Lisa Pokorny, Charlotte Quigley, Philip Thomas, Juan Carlos Villarreal, Megan M. Augustin, Matthew D. Barrett, Regina S. Baucom, David J. Beerling, Ruben Maximilian Benstein, Ed Biffin, Samuel F. Brockington, Dylan O. Burge, Jason N. Burris, Kellie P. Burris, Valérie Burtet-Sarramegna, Ana L. Caicedo, Steven B. Cannon, Zehra Çebi, Ying Chang, Caspar Chater, John M. Cheeseman, Tao Chen, Neil D. Clarke, Harmony Clayton, Sarah Covshoff, Barbara J. Crandall-Stotler, Hugh Cross, Claude W. dePamphilis, Joshua P. Der, Ron Determann, Rowan C. Dickson, Verónica S. Di Stilio, Shona Ellis, Eva Fast, Nicole Feja, Katie J. Field, Dmitry A. Filatov, Patrick M. Finnegan, Sandra K. Floyd, Bruno Fogliani, Nicolás García, Gildas Gâteblé, Grant T. Godden, Falicia (Qi Yun) Goh, Stephan Greiner, Alex Harkess, James Mike Heaney, Katherine E. Helliwell, Karolina Heyduk, Julian M. Hibberd, Richard G. J. Hodel, Peter M. Hollingsworth, Marc T. J. Johnson, Ricarda Jost, Blake Joyce, Maxim V. Kapralov, Elena Kazamia, Elizabeth A. Kellogg, Marcus A. Koch, Matt Von Konrat, Kálmán Könyves, Toni M. Kutchan, Vivienne Lam, Anders Larsson, Andrew R. Leitch, Roswitha Lentz, Fay-Wei Li, Andrew J. Lowe, Martha Ludwig, Paul S. Manos, Evgeny Mavrodiev, Melissa K. McCormick, Michael McKain, Tracy McLellan, Joel R. McNeal, Richard E. Miller, Matthew N. Nelson, Yanhui Peng, Paula Ralph, Daniel Real, Chance W. Riggins, Markus Ruhsam, Rowan F. Sage, Ann K. Sakai, Moira Scascitella, Edward E. Schilling, Eva-Marie Schlösser, Heike Sederoff, Stein Servick, Emily B. Sessa, A. Jonathan Shaw, Shane W. Shaw, Erin M. Sigel, Cynthia Skema, Alison G. Smith, Ann Smithson, C. Neal Stewart, John R. Stinchcombe, Peter Szövényi, Jennifer A. Tate, Helga Tiebel, Dorset Trapnell, Matthieu Villegente, Chun-Neng Wang, Stephen G. Weller, Michael Wenzel, Stina Weststrand, James H. Westwood, Dennis F. Whigham, Shuangxiu Wu, Adrien S. Wulff, Yu Yang, Dan Zhu, Cuili Zhuang, Jennifer Zuidof, Mark W. Chase, J. Chris Pires, Carl J. Rothfels, Jun Yu, Cui Chen, Li Chen, Shifeng Cheng, Juanjuan Li, Ran Li, Xia Li, Haorong Lu, Yanxiang Ou, Xiao Sun, Xuemei Tan, Jingbo Tang, Zhijian Tian, Feng Wang, Jun Wang, Xiaofeng Wei, Xun Xu, Zhixiang Yan, Fan Yang, Xiaoni Zhong, Feiyu Zhou, Ying Zhu, Yong Zhang, Saravanaraj Ayyampalayam, Todd J. Barkman, Nam-phuong Nguyen, Naim Matasci, David R. Nelson, Erfan Sayyari, Eric K. Wafula, Ramona L. Walls, Tandy Warnow, Hong An, Nils Arrigo, Anthony E. Baniaga, Sally Galuska, Stacy A. Jorgensen, Thomas I. Kidder, Hanghui Kong, Patricia Lu-Irving, Hannah E. Marx, Xinshuai Qi, Chris R. Reardon, Brittany L. Sutherland, George P. Tiley, Shana R. Welles, Rongpei Yu, Shing Zhan, Lydia Gramzow, Günter Theißen, Gane Ka-Shu Wong

**Affiliations:** 1 Department of Plant Biology, University of Georgia, Athens, GA USA; 2 Department of Ecology and Evolutionary Biology, University of Arizona, Tucson, AZ USA; 3 Department of Biological Sciences, University of Alberta, Edmonton, Alberta Canada; 4 Department of Biology, The University of British Columbia Okanagan, Kelowna, British Columbia Canada; 5 Department of Biology, University of Florida, Gainesville, FL USA; 6 Florida Museum of Natural History, University of Florida, Gainesville, FL USA; 7 Department of Botany, University of British Columbia, Vancouver, British Columbia Canada; 8 German Centre for Integrative Biodiversity Research (iDiv), Halle-Jena-Leipzig, Germany; 9 Botanical Institute, University of Cologne, Cologne, Germany; 10 Department of Electrical and Computer Engineering, University of California, San Diego, San Diego, CA USA; 11 Institute of Computer Science, Martin Luther University Halle-Wittenberg, Halle (Saale), Germany; 12 Institute of Agricultural and Nutritional Sciences, Martin Luther University Halle-Wittenberg, Halle (Saale), Germany; 13 BIOSS Centre for Biological Signalling Studies, University of Freiburg, Freiburg, Germany; 14 Plant Cell Biology, Faculty of Biology, University of Marburg, Marburg, Germany; 15 UF Biodiversity Institute, and UF Genetics Institute, University of Florida, Gainesville, FL USA; 16 New York Botanical Garden, New York, NY USA; 17 Department of Evolutionary Genetics, Max Planck Institute for Evolutionary Biology, Plön, Germany; 18 Negaunee Institute for Plant Conservation Science and Action, Chicago Botanic Garden, Glencoe, IL USA; 19 Program in Plant Biology and Conservation, Northwestern University, Evanston, IL USA; 20 Department of Horticulture, Michigan State University, East Lansing, MI USA; 21 Department of Botany, University of Wisconsin-Madison, Madison, WI USA; 22 Ocean University of China, Qingdao, China; 23 Department of Biological Sciences, University of North Texas, Denton, TX USA; 24 Centre for Plant Biotechnology and Genomics (CBGP, UPM-INIA), Madrid, Spain; 25 Department of Biodiversity and Conservation, Real Jardín Botánico (RJB-CSIC), Madrid, Spain; 26 Jodrell Laboratory, Royal Botanic Gardens, Kew, London, UK; 27 School of Marine Sciences, University of Maine, Orono, ME USA; 28 Royal Botanic Garden Edinburgh, Edinburgh, UK; 29 Department of Plant Biology, Laval University, Quebec, Quebec, Canada; 30 Donald Danforth Plant Science Center, St Louis, MO USA; 31 School of Biological Sciences, The University of Western Australia, Perth, Western Australia Australia; 32 Kings Park and Botanic Garden, Department of Biodiversity, Conservation and Attractions, Perth, Western Australia Australia; 33 Australian Tropical Herbarium, James Cook University, Cairns, Queensland Australia; 34 Department of Ecology and Evolutionary Biology, University of Michigan, Ann Arbor, MI USA; 35 Department of Animal and Plant Sciences, University of Sheffield, Sheffield, UK; 36 Umeå Plant Science Centre, Umeå Universitet, Umeå, Sweden; 37 Australian Centre for Evolutionary Biology and Biodiversity, Environment Institute, School of Earth and Environmental Science, University of Adelaide, Adelaide, South Australia Australia; 38 Department of Plant Sciences, University of Cambridge, Cambridge, UK; 39 Royal Botanic Garden Sydney, Sydney, New South Wales, Australia; 40 Department of Plant Sciences, University of Tennessee, Knoxville, TN USA; 41 Center for Agricultural Synthetic Biology, University of Tennessee, Knoxville, TN USA; 42 Department of Food Science, University of Tennessee, Knoxville, TN USA; 43 Department of Food, Bioprocessing and Nutrition Sciences, North Carolina State University, Raleigh, NC USA; 44 Institute for Exact and Applied Sciences, University of New Caledonia, Noumea, New Caledonia; 45 Department of Biology, University of Massachusetts, Amherst, MA USA; 46 USDA-Agricultural Research Service, Corn Insects and Crop Genetics Research Unit, Ames, IA USA; 47 Department of Botany and Plant Pathology, Oregon State University, Corvallis, OR USA; 48 Department of Molecular Biology and Biotechnology, University of Sheffield, Sheffield, UK; 49 Department of Plant Biology, University of Illinois, Urbana-Champaign, Urbana, IL USA; 50 Fairy Lake Botanical Garden, Chinese Academy of Sciences, Shenzhen, China; 51 Yale-NUS College, Singapore, Republic of Singapore; 52 School of Molecular Sciences, The University of Western Australia, Perth, Western Australia Australia; 53 Department of Plant Biology, Southern Illinois University, Carbondale, IL USA; 54 Department of Anatomy, University of Otago, Dunedin, New Zealand; 55 Biology Department, Pennsylvania State University, University Park, PA USA; 56 Department of Biological Science, California State University Fullerton, Fullerton, CA USA; 57 Atlanta Botanical Garden, Atlanta, GA USA; 58 Massey University, School of Fundamental Sciences, Palmerston North, New Zealand; 59 Department of Biology, University of Washington, Seattle, WA USA; 60 Centre for Plant Sciences, Faculty of Biological Sciences, University of Leeds, Leeds, UK; 61 Department of Plant Sciences, University of Oxford, Oxford, UK; 62 School of Biological Sciences, Monash University, Melbourne, Victoria, Australia; 63 Institut Agronomique néo-Calédonien (IAC), Equipe ARBOREAL, Païta, New Caledonia; 64 Facultad de Ciencias Forestales y de la Conservación de la Naturaleza, Universidad de Chile, Santiago, Chile; 65 Genome Institute of Singapore, Singapore, Singapore; 66 Max Planck Institute of Molecular Plant Physiology, Potsdam-Golm, Germany; 67 Biosciences, College of Life and Environmental Sciences, University of Exeter, Exeter, UK; 68 Marine Biological Association, The Laboratory, Plymouth, UK; 69 Department of Ecology and Evolutionary Biology, Yale University, New Haven, CT USA; 70 Department of Biology, University of Toronto Mississauga, Mississauga, Ontario Canada; 71 School of Life Sciences, La Trobe University, Bundoora, Victoria Australia; 72 CyVerse, BIO5 Institute, University of Arizona, Tucson, AZ USA; 73 School of Natural and Environmental Sciences, Newcastle University, Newcastle upon Tyne, UK; 74 University of Missouri, St Louis, St Louis, MO USA; 75 Centre for Organismal Studies Heidelberg, Department of Biodiversity and Plant Systematics, Botanic Garden and Herbarium Heidelberg, University of Heidelberg, Heidelberg, Germany; 76 The Field Museum, Chicago, IL USA; 77 Royal Horticultural Society Garden Wisley, Woking, UK; 78 University of Reading Herbarium, School of Biological Sciences, University of Reading, Reading, UK; 79 Department of Pharmaceutical Biosciences, Uppsala University, Uppsala, Sweden; 80 School of Biological and Chemical Sciences, Queen Mary University of London, London, UK; 81 Boyce Thompson Institute, Cornell University, Ithaca, NY USA; 82 Environment Institute, School of Biological Science, University of Adelaide, Adelaide, South Australia Australia; 83 Department of Biology, Duke University, Durham, NC USA; 84 Smithsonian Environmental Research Center, Edgewater, MD USA; 85 Department of Biological Sciences, University of Alabama, Tuscaloosa, AL USA; 86 School of Molecular and Cell Biology, University of the Witwatersrand, Johannesburg, South Africa; 87 Department of Ecology, Evolution and Organismal Biology, Kennesaw State University, Kennesaw, GA USA; 88 Flower Diversity Institute, Arvada, CO USA; 89 CSIRO Agriculture and Food, Perth, Western Australia Australia; 90 Millennium Seed Bank, Wakehurst, Royal Botanic Gardens, Kew, Ardingly UK; 91 The UWA Institute of Agriculture, The University of Western Australia, Perth, Western Australia Australia; 92 Centers for Disease Control and Prevention, Atlanta, GA USA; 93 Department of Primary Industries and Regional Development, Perth, Western Australia Australia; 94 Department of Crop Sciences, University of Illinois at Urbana-Champaign, Urbana, IL USA; 95 Department of Ecology and Evolutionary Biology, The University of Toronto, Ontario, Canada; 96 Department of Ecology and Evolutionary Biology, University of California, Irvine, Irvine, CA USA; 97 Department of Ecology and Evolutionary Biology, University of Tennessee, Knoxville, TN USA; 98 Department of Plant and Microbial Biology, North Carolina State University, Raleigh, NC USA; 99 Manoa, Honolulu, HI USA; 100 Department of Biology, University of Louisiana at Lafayette, Lafayette, LA USA; 101 Morris Arboretum of the University of Pennsylvania, Philadelphia, PA USA; 102 Koffler Scientific Reserve, University of Toronto, King City, Ontario Canada; 103 Department of Systematic and Evolutionary Botany, University of Zurich, Zurich, Switzerland; 104 National Taiwan University, Institute of Ecology and Evolutionary Biology, Department of Life Science, Taipei, Taiwan; 105 Systematic Biology, Department of Organismal Biology, Evolutionary Biology Centre, Uppsala University, Uppsala, Sweden; 106 Department of Plant Pathology, Physiology and Weed Science, Virginia Tech, Blacksburg, VA USA; 107 CAS Key Laboratory of Genome Sciences and Information, Beijing Key Laboratory of Genome and Precision Medicine Technologies, Beijing Institute of Genomics, Chinese Academy of Sciences, Beijing, China; 108 Key Laboratory of Agricultural Biological Functional Genes, Northeast Agricultural University, Harbin, China; 109 College of Life Science, Qingdao Agricultural University, Qingdao, China; 110 Agriculture and Agri-Food Canada, Lacombe, Alberta Canada; 111 Department of Environment and Agriculture, Curtin University, Bentley, Western Australia Australia; 112 Bond Life Sciences Center, Division of Biological Sciences, University of Missouri, Columbia, MO USA; 113 Department of Zoology, University of British Columbia, Vancouver, British Columbia Canada; 114 University Herbarium and Department of Integrative Biology, University of California, Berkeley, Berkeley, CA USA; 115 Beijing Genomics Institute-Wuhan, Wuhan, China; 116 BGI-Shenzhen, Shenzhen, China; 117 Agricultural Genome Institute at Shenzhen, Chinese Academy of Agricultural Sciences, Shenzhen, China; 118 Huahan Gene, Shenzhen, China; 119 MGI, BGI-Shenzhen, Shenzhen, China; 120 Allwegene Technology, Beijing, China; 121 iCarbonX, Shenzhen, China; 122 Georgia Advanced Computing Resource Center, University of Georgia, Athens, GA USA; 123 Department of Biological Sciences, Western Michigan University, Kalamazoo, MI USA; 124 Department of Computer Science and Engineering, University of California, San Diego, San Diego, CA USA; 125 Lawrence J. Ellison Institute for Transformative Medicine, University of Southern California, Los Angeles, CA USA; 126 Microbiology, Immunology and Biochemistry, The University of Tennessee Health Science Center, Memphis, TN USA; 127 Department of Computer Science, University of Illinois, Urbana-Champaign, Urbana, IL USA; 128 Division of Biological Sciences, University of Missouri, Columbia, MO USA; 129 Arizona Research Laboratories, University of Arizona, Tucson, AZ USA; 130 Key Laboratory of Plant Resources Conservation and Sustainable Utilization, South China Botanical Garden, Chinese Academy of Sciences, Guangzhou, China; 131 Flower Research Institute, Yunnan Academy of Agricultural Sciences, Kunming, China; 132 Department of Genetics, Matthias Schleiden Institute, Friedrich-Schiller-University Jena, Jena, Germany; 133 Department of Medicine, University of Alberta, Edmonton, Alberta Canada

**Keywords:** Molecular evolution, Adaptive radiation, Phylogenomics, Plant evolution

## Abstract

Green plants (Viridiplantae) include around 450,000–500,000 species^1,2^ of great diversity and have important roles in terrestrial and aquatic ecosystems. Here, as part of the One Thousand Plant Transcriptomes Initiative, we sequenced the vegetative transcriptomes of 1,124 species that span the diversity of plants in a broad sense (Archaeplastida), including green plants (Viridiplantae), glaucophytes (Glaucophyta) and red algae (Rhodophyta). Our analysis provides a robust phylogenomic framework for examining the evolution of green plants. Most inferred species relationships are well supported across multiple species tree and supermatrix analyses, but discordance among plastid and nuclear gene trees at a few important nodes highlights the complexity of plant genome evolution, including polyploidy, periods of rapid speciation, and extinction. Incomplete sorting of ancestral variation, polyploidization and massive expansions of gene families punctuate the evolutionary history of green plants. Notably, we find that large expansions of gene families preceded the origins of green plants, land plants and vascular plants, whereas whole-genome duplications are inferred to have occurred repeatedly throughout the evolution of flowering plants and ferns. The increasing availability of high-quality plant genome sequences and advances in functional genomics are enabling research on genome evolution across the green tree of life.

## Abstract (web summary)

The One Thousand Plant Transcriptomes Initiative provides a robust phylogenomic framework for examining green plant evolution that comprises the transcriptomes and genomes of diverse species of green plants.

## Main

**Fig. 1:**
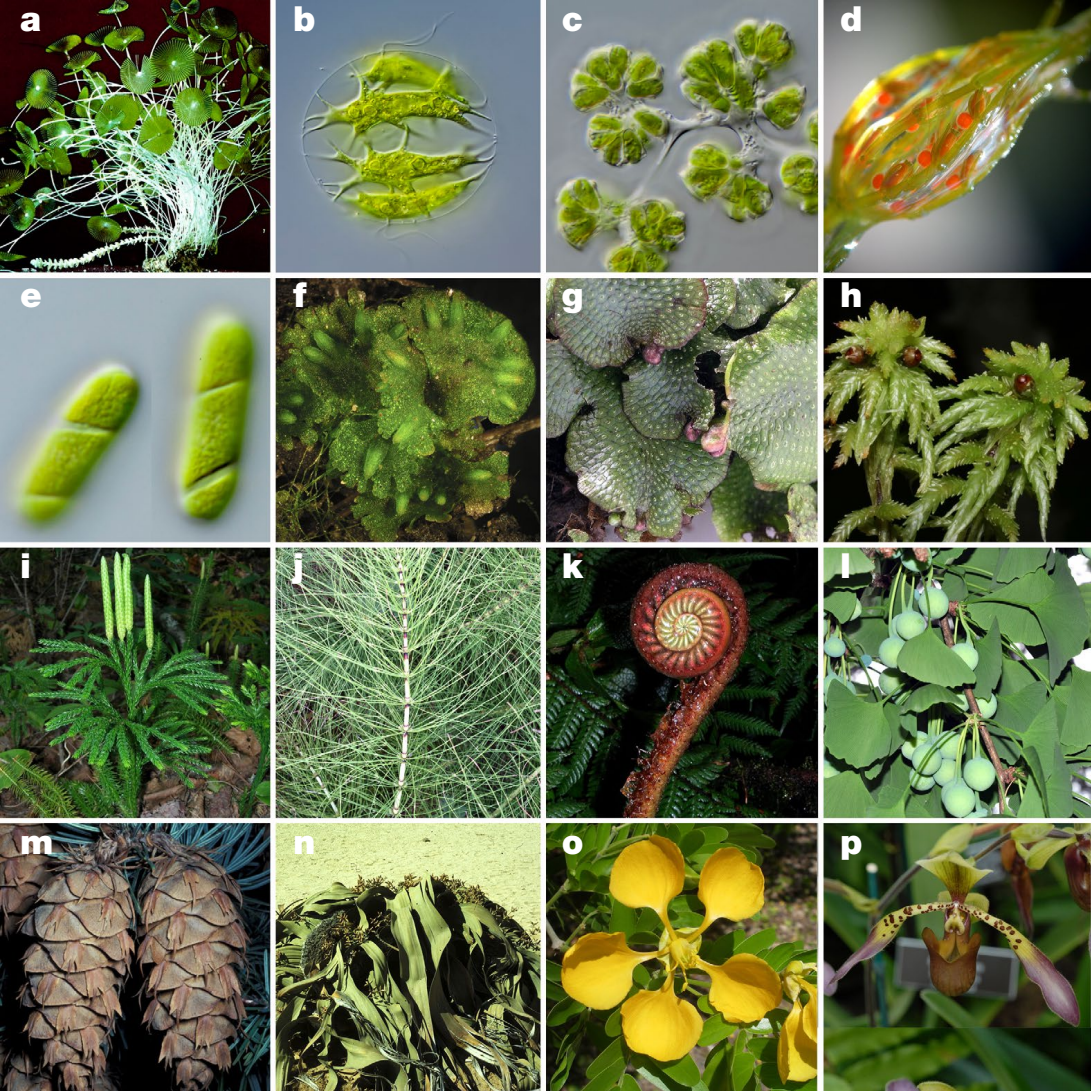
Diversity within the Viridiplantae. **a**–**e**, Green algae. **a**, *Acetabularia* sp. (Ulvophyceae). **b**, *Stephanosphaera pluvialis* (Chlorophyceae). **c**, *Botryococcus* sp. (Trebouxiophyceae). **d**, *Chara* sp. (Charophyceae). **e**, ‘*Spirotaenia*’ sp. (taxonomy under review) (Zygnematophyceae). **f**–**p**, Land plants. **f**, *Notothylas orbicularis* (Anthocerotophyta (hornwort)). **g**, *Conocephalum conicum* (Marchantiophyta (thalloid liverwort)). **h**, *Sphagnum* sp. (Bryophyta (moss)). **i**, *Dendrolycopodium obscurum* (Lycopodiophyta (club moss)). **j**, *Equisetum telmateia* (Polypodiopsida, Equisetidae (horsetail)). **k**, *Parablechnum schiedeanum* (Polypodiopsida, Polypodiidae (leptosporangiate fern)). **l**, *Ginkgo biloba* (Ginkgophyta). **m**, *Pseudotsuga menziesii* (Pinophyta (conifer)). **n**, *Welwitschia mirabilis* (Gnetophyta). **o**, *Bulnesia arborea* (Angiospermae, eudicot, rosid). **p**, *Paphiopedilum lowii* (Angiospermae, monocot, orchid). **a**, Photograph reproduced with permission of Thieme Verlag, Stuttgart^66^. **b**–**e**, Photographs courtesy of M. Melkonian. **f**–**j**, **l**–**n**, **p**, Photographs courtesy of D.W.S. **k**, Photograph courtesy of R. Moran. **o**, Photograph courtesy of W. Judd.

Viridiplantae comprise an estimated 450,000–500,000 species^1,2^, encompass a high level of diversity and evolutionary timescales^3^, and have important roles in all terrestrial and most aquatic ecosystems. This ecological diversity derives from developmental, morphological and physiological innovations that enabled the colonization and exploitation of novel and emergent habitats. These innovations include multicellularity and the development of the plant cuticle, protected embryos, stomata, vascular tissue, roots, ovules and seeds, and flowers and fruit (Fig. 1). Thus, plant evolution ultimately influenced environments globally and created a cascade of diversity in other lineages that span the tree of life. Plant diversity has also fuelled agricultural innovations and growth in the human population^4^.

Phylogenomic approaches are now widely used to resolve species relationships^5^ as well as the evolution of genomes, gene families and gene function^6^. We used mostly vegetative transcriptomes for a broad taxonomic sampling of 1,124 species together with 31 published genomes to infer species relationships and characterize the relative timing of organismal, molecular and functional diversification across green plants.

**Fig. 2:**
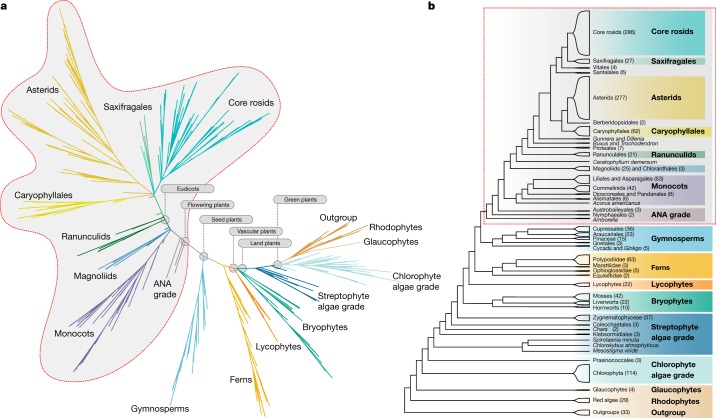
Phylogenetic inferences of major clades. Phylogenetic inferences were based on ASTRAL analysis of 410 single-copy nuclear gene families extracted from genome and transcriptome data from 1,153 species, including 1,090 green plant (Viridiplantae) species (Supplementary Table 1). **a**, Phylogram showing internal branch lengths proportional to coalescent units (2*N*_e_ generations) between branching events, as estimated by ASTRAL-II^15^ v.5.0.3. **b**, Relationships among major clades with red box outlining flowering plant clade. Species numbers are shown for each lineage. Most inferred relationships were robust across data types and analyses (Supplementary Figs. 1–3) with some exceptions (Supplementary Fig. 6). Data and analysis scripts are available at 10.5281/zenodo.3255100.

We evaluated gene history discordance among single-copy genes. This is expected in the face of rapid species diversification, owing to incomplete sorting of ancestral variation between speciation events^7^. Hybridization^8^, horizontal gene transfer^9^, gene loss following gene and genome duplications^10^ and estimation error can also contribute to gene-tree discordance. Nevertheless, through rigorous gene and species tree analyses, we derived robust species tree estimates (Fig. 2 and Supplementary Figs. 1–3). Gene-family expansions and genome duplications are recognized sources of variation for the evolution of gene function and biological innovations^11,12^. We inferred the timing of ancient genome duplications and large gene-family expansions. Our findings suggest that extensive gene-family expansions or genome duplications preceded the evolution of major innovations in the history of green plants.

## Integrated analysis of genome evolution

**Fig. 3:**
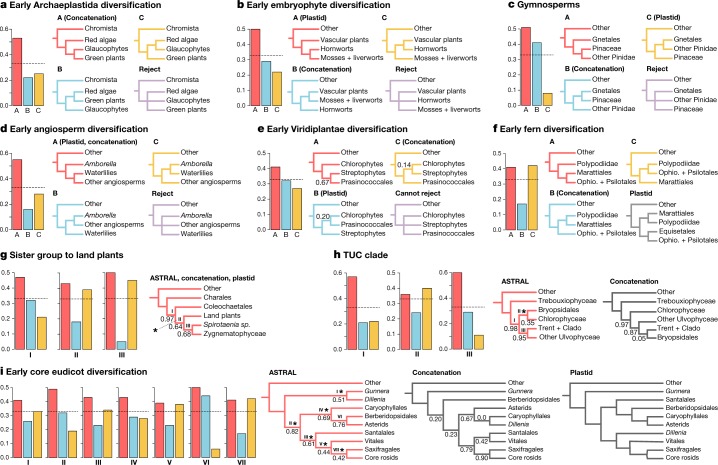
Alternative branching orders for contentious relationships. Local posterior probabilities (shown only when below 1.0) and gene-tree quartet frequencies (bar graphs) for alternative branching orders for contentious relationships in the plant phylogeny (see text). **a**, Early Archaeplastida diversification. **b**, Early embryophyte diversification. **c**, Gymnosperms. **d**, Early angiosperm diversification. **e**, Early Viridiplantae diversification. **f**, Early fern diversification. **g**, The sister lineage to land plants. **h**, Trebouxiophyceae, Ulvophyceae and Chlorophyceae. **i**, Eudicot diversification. Red bars represent the ASTRAL topology; blue and yellow trees and bars represent the frequencies of alternative branching orders in ASTRAL. The topologies recovered in the concatenated supermatrix analysis and plastid gene analyses are also indicated. Dashed horizontal lines mark expectation for a hard polytomy (purple). In **g**–**i**, panels include more than 4 tips, so nodes are delineated with Roman numerals and bar graphs are shown for each node and asterisks above branches indicate failure to reject the hypothesis that the node is a polytomy. Data and analysis scripts are available at 10.5281/zenodo.3255100.

Because genome sizes vary by 2,340-fold in land plants^13^ and 4,680-fold in chlorophyte and streptophyte green algae^14^, we used a reduced-representation sequencing approach to reconstruct gene and species histories. Specifically, we generated 1,342 transcriptomes representing 1,124 species across Archaeplastida, including green plants, glaucophytes and red algae. Comparing phylogenetic inferences based on nuclear and plastid genes (Figs. 2, 3 and Supplementary Figs. 1–3), we obtained well-supported, largely congruent results across diverse datasets and analyses. Resolution of some relationships, however, was confounded by gene-tree discordance (Fig. 3), which is attributable to factors that include rapid diversification, reticulate evolution, gene duplication and loss, and estimation error.

**Fig. 4:**
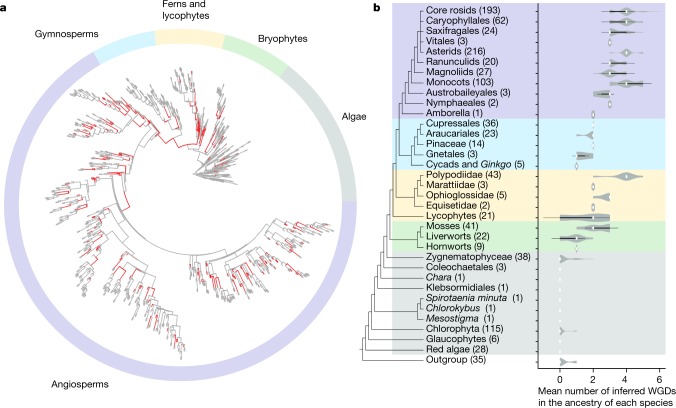
The distribution of inferred ancient WGDs across lineages of green plants. **a**, The locations of estimated WGDs are labelled red in the phylogeny of all 1000 Plants (1KP) samples. **b**, The number of inferred ancient polyploidization events within each lineage is shown in the violin plots. The white dot indicates the median, the thick black bars represent the interquartile range, the thin black lines define the 95% confidence interval and the grey shading represents the density of data points. The sample sizes for each lineage are shown within parentheses along with taxon names on the phylogeny. The phylogenetic placement of inferred WGDs is illustrated in Supplementary Fig. 8 and data supporting each WGD inference are provided in Supplementary Table 2.

Inferred whole-genome duplications (WGDs; that is, polyploidy) across the gene-tree summary phylogeny estimated using ASTRAL^15^ were not uniformly distributed (Fig. 4, Supplementary Fig. 8 and Supplementary Table 2). Comparing distributions of gene duplication times for each species^16^ (Supplementary Table 3) and orthologue divergence times^17^ (Supplementary Table 4) with gene-tree analyses^18^ (Supplementary Tables 5, 6), we inferred 244 ancient WGDs across Viridiplantae (Supplementary Fig. 8 and Supplementary Table 2). Although there are limitations to the inference of WGD events using this approach, we found that comparisons of these results with 65 overlapping published genome-based WGD inferences revealed 6 false-negative results in our tree-based estimates and no false-positive results (Supplementary Table 2). Analyses based on whole-genome sequences are needed for further resolution of WGD events.

With the exception of most *Selaginella* species and some liverworts (Fig. 1g), our analyses implicated at least one ancient WGD in the ancestry of every land plant lineage. By contrast, most algal lineages showed no evidence of WGD. Notably, the predicted sister clade of land plants (Fig. 2), Zygnematophyceae (Fig. 1e), exhibited the highest density of WGDs among algal lineages (Fig. 4), although the apparent increase in WGD was largely restricted to the desmid clade (Desmidiales) within Zygnematophyceae.

Increased diversification rates did not precisely co-occur with WGDs on the phylogeny. WGDs are expected to contribute to the evolution of novel gene function^11,12^. For example, novel functions among duplicate MADS-box genes that arose through WGD have been linked to the origin of flowering plants^19,20^ and core eudicots^21^, and functional diversification of gene families after WGD has contributed to the evolution of fruit colour in tomato species^22,23^ and to nodule development within legumes^22,24^. Consistent with previous studies with less extensive taxon sampling^24–27^, however, we inferred lags between WGDs and increased species diversity. Integrated phylogenomic and functional investigations are required to gain a mechanistic understanding of the lag between WGD, the evolution of novel gene functions and their potential influence on diversification rates.

**Fig. 5:**
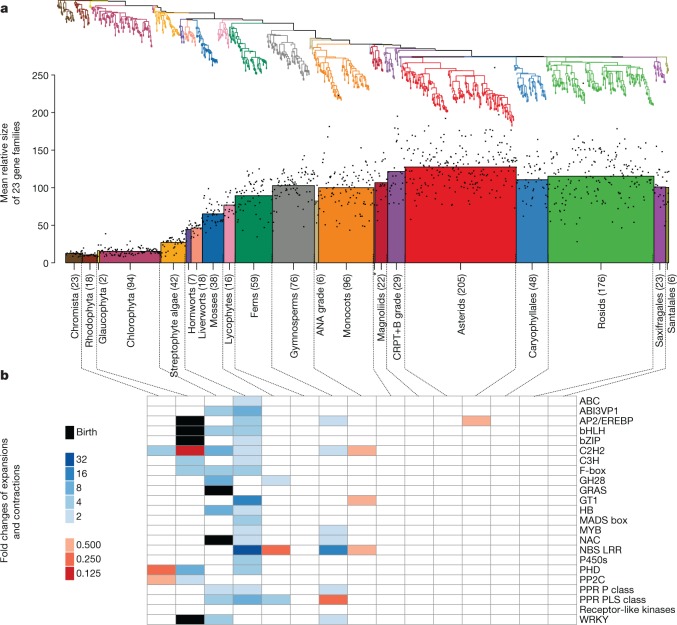
Assessment of significant expansions and contractions of largest plant gene families. **a**, Weighted average gene-family size for species groups (normalized to account for differences in gene-family sizes, weight = 1/(maximum observed gene-family size)). The ANA grade comprises Amborellales, Nymphaeales and Austrobaileyales, successive sister lineages to a clade with the remaining extant angiosperms; the ‘CRPT+B’ grade includes Ceratophyllales, Ranunculales, Proteales lineages and a Trochodendrales + Buxales clade in the ASTRAL tree (Fig. 2). Sample sizes are proportional to bar widths (from left to right, *n* = 23 (Chromista), 18 (Rhodophyta), 2 (Glaucophyta), 94 (Chlorophyta), 42 (streptophyte algae), 7 (hornworts), 18 (liverworts), 38 (mosses), 16 (lycophytes), 59 (ferns; monilophytes), 76(gymnosperms), 6 (ANA grade), 96 (monocots), 1 (*representing Chloranthales), 22 (magnoliids), 29 (CRPT+B grade), 205 (asterids), 48 (caryophyllids), 176 (rosids), 23 (Saxifragales) and 6 (Santalales). **b**, Gene families exhibiting significant copy number changes (two-sided Kolmogorov–Smirnov test; *P* < 1 × 10^−6^; gene-family expansions represent a gain of more than 50% and contractions represent a loss of more than 33%) with colour codes showing the magnitude of the observed fold changes. Data and analysis scripts are available at https://github.com/GrosseLab/OneKP-gene-family-evo.

Gene-family expansions (and contractions) contribute to the dynamic evolution of metabolic, regulatory and signalling networks^28,29^. Given the inherent limitations of transcriptome data, we searched for large-fold changes in 23 of the largest gene families in *Arabidopsis thaliana*^30^ that are involved in many important functions (such as transcriptional regulation, enzymatic and signalling function, and transport; Fig. 5 and Supplementary Tables 7, 8). Although our RNA-sequencing-based sampling of expressed genes is incomplete, the median representation of universally conserved genes^31^ was 80–90% for taxa across Viridiplantae (Extended Data Fig. 3a, b). Furthermore, there was a strong correlation (*r* = 0.95) between gene-family sizes in our transcriptomes (focusing on the largest gene families) and those of fully sequenced genomes (Extended Data Fig. 3c–f). We identified gene-family expansions and contractions, including some that have been described previously^32–34^. Specifically, the *AP2*, *bHLH*, *bZip* and *WRKY* transcription factor families were inferred to be present in the last common ancestor of Viridiplantae, whereas the origin of *GRAS* and *NAC* genes occurred in early streptophytes after divergence from the chlorophyte algal lineage (Fig. 5). The highest concentration of expansion events was inferred along the ‘spine’ of the phylogeny between the origins of Viridiplantae and vascular plants (Fig. 5b and Supplementary Table 7). Expansions of some focal gene families also continued after the origin of embryophytes; however, no expansions occurred in association with the origin and radiation of angiosperms (Fig. 5). Gene-family expansions and functional diversification may have contributed to the adaptations required for life in terrestrial habitats, but the sizes of these focal gene families apparently stabilized in the face of continued gene duplication and loss throughout the evolution of vascular plants.

## Primary acquisition of the plastid

The primary acquisition of the plastid in an ancestor of extant Archaeplastida was a pivotal event in the history of life. All possible relationships among Viridiplantae, Glaucophyta and Rhodophyta have been hypothesized, with alternative implications for the gain and loss of characters^35^ in the early history of the three lineages. Strong support for the sister relationship of Viridiplantae and Glaucophyta^35^ (Figs. 2, 3a) found here indicates that ancestral red algae lost flagella and peptidoglycan biosynthesis, perhaps associated with a reduction in genome size^36^. Peptidoglycan biosynthesis was independently lost early in the evolution of Chlorophyta^37^ and within angiosperms^38^.

## The history of Viridiplantae

The origin of Viridiplantae is marked by the loss of light-harvesting phycobilisomes composed of phycobiliproteins, the evolution of the accessory photosynthetic pigment chlorophyll *b*, which has a distinct light-absorption spectrum relative to chlorophyll *a*, and intraplastidial starch synthesis and deposition. Viridiplantae are consistently recovered as monophyletic, with early diverging Chlorophyta and Streptophyta lineages^39–41^. However, the placement of the picoplanktonic algal lineage Prasinococcales was unstable in our analyses (Fig. 3e).

### Diversification within Chlorophyta

All nuclear-gene analyses resolved a grade of largely marine unicellular lineages subtending the core clade consisting of Trebouxiophyceae, Ulvophyceae and Chlorophyceae^42^ (Fig. 1a–c and Supplementary Figs. 1–3). The nuclear supermatrix and ASTRAL trees placed Trebouxiophyceae as sister to a clade containing Chlorophyceae and Ulvophyceae^42,43^. However, whereas the supermatrix trees supported Ulvophyceae as monophyletic, the ASTRAL tree resolved Ulvophyceae as a grade and Bryopsidales is poorly supported as sister to Chlorophyceae (Fig. 3h). All tree estimates suggest that there were multiple origins of multicellularity within Ulvophyceae. Only 12 out of 119 sampled chlorophyte species exhibited evidence of a WGD in their ancestry, and most of these putative WGDs were restricted to single clades.

### Streptophyta

The evolution of streptophytes was associated with several adaptations to terrestrial habitats^44–46^. All analyses recovered *Mesostigma*, *Chlorokybus* and *Spirotaenia minuta* in a clade that is sister to the remainder of Streptophyta^39^ with successive divergence of Klebsormidiales, Charophyceae (Fig. 1d), Coleochaetophyceae and Zygnematophyceae (Fig. 1e) relative to Embryophyta. However, with greatly increased taxon sampling relative to our previous work^39^, internal branch lengths are diminished, and we could not reject the possibility of a true radiation giving rise to Coleochaetales, Zygnematophyceae and embryophyte lineages (land plants; Figs. 1f–p, 3g(II)). Although quartet support for a clade of Coleochaetales and Zygnematophyceae as sister to embryophytes was similar to support for Zygnematophyceae as sister to embryophytes, a clade consisting of Coleochaetales and land plants was not supported.

### Embryophyta

Land plants include many of the most familiar green plants (for example, bryophytes (Fig. 1f–h), lycophytes (Fig. 1i), ferns (Fig. 1j, k) and seed plants (Fig. 1l–p)). They exhibit key innovations, including protected reproductive organs (archegonia and antheridia) and the development of the zygote within an archegonium into an embryo that receives maternal nutrition. Resolving relationships among bryophytes (mosses, liverworts and hornworts) and their relationships to the remaining land plants has long been problematic, but is critical for understanding the evolution of fundamental innovations within land plants, including the tolerance to desiccation, shifts in the dominance of multicellular haploid and diploid generations, and parental retention of a multicellular embryo.

Bryophytes have sometimes been resolved as a grade^47,48^, with liverworts, mosses and hornworts as successive sister groups to Tracheophyta (vascular plants; Fig. 1i–p). We recovered extant bryophytes as monophyletic in the ASTRAL analysis of nuclear gene trees (Fig. 3b) and plastome analyses, with hornworts sister to a moss and liverwort clade. All analyses rejected the hypothesis that liverworts are sister to all other extant land plant lineages^39,49^.

The largest number of gene-family expansions in our analyses was associated with the origin of land plants and the evolution of bryophytes (transition between streptophyte algae and bryophytes in Fig. 5b). By contrast, we found no evidence of WGD on the stem branch for land plants (Supplementary Tables 5, 6).

### Vascular plants

Within the vascular plants, lycophytes are supported as the sister group of Euphyllophyta (ferns and seed plants). We found no evidence of pan-vascular-plant or ancestral euphyllophyte WGDs, but some gene-family expansions were associated with the origin of vascular plants (Fig. 5b).

Within ferns (Polypodiopsida), plastid data weakly support Equisetales as sister to Psilotales and Ophioglossales (Supplementary Fig. 3), whereas nuclear gene analyses robustly place Equisetales sister to the remaining ferns^50^. The supermatrix and plastome-based trees placed Marattiales sister to the leptosporangiate ferns^50^ (Polypodiidae), but ASTRAL recovers nearly equal quartet support for this hypothesis or for Marattiales as sister to Psilotales and Ophioglossales (Fig. 3f). Leptosporangiate ferns (Fig. 1k) experienced more WGD events than any other lineage of Viridiplantae outside the angiosperms, with an average of 3.79 inferred WGDs in the history of each sampled species (Fig. 4). WGD was inferred in an ancestor of all extant ferns and an additional 19 putative WGDs were implicated in the ancestry of fern subclades (Ophioglossaceae and Polypodiaceae; Fig. 4, Supplementary Fig. 8 and Supplementary Tables 2, 5, 6). Considering the high chromosome numbers of some ferns, our discovery that they exhibit one of the highest frequencies of palaeopolyploidization among green plants is not unexpected^51^.

**Extended Data Fig. 1:**
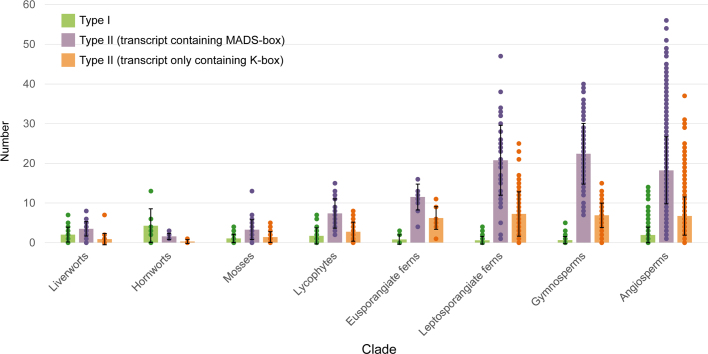
Mean number of MADS-box genes in the transcriptomes of different plant clades. Type I genes are shown in green; type II genes are shown in purple and orange. Transcripts in which only a K-box was identified (which are probably partial transcripts of type II genes) are shown in orange. Data are mean ± s.d. Dots indicate the numbers of MADS-box genes in individual transcriptomes. Sample sizes (*n*) are as follows: liverworts, *n* = 26; hornworts, *n* = 7; mosses, *n* = 37; lycophytes, *n* = 22; eusporangiate ferns, *n* = 10; leptosporangiate ferns, *n* = 62; gymnosperms, *n* = 84; and angiosperms, *n* = 820. A total of 1,068 transcriptomes were analysed for this figure.

**Extended Data Fig. 2:**
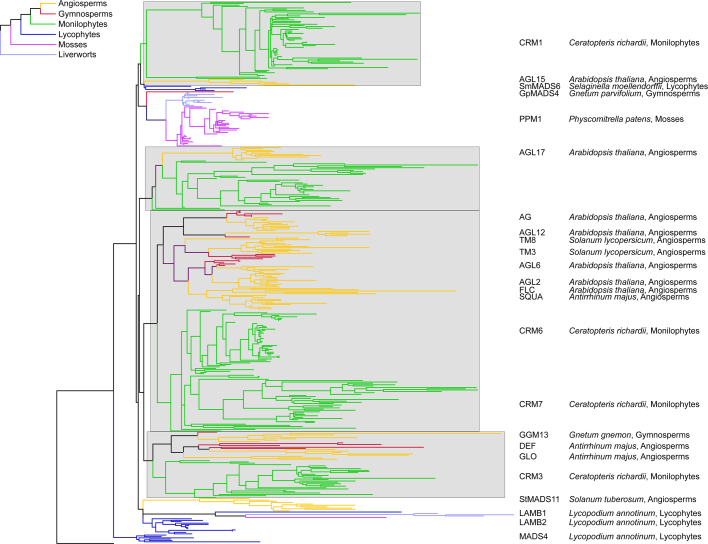
RAxML phylogeny of classic type II MIKC^c^ MADS-box genes of liverworts, mosses, lycophytes, monilophytes (ferns) and spermatophytes (seed plants). *Cg*MADS1 from *Chara globularis* was used as a representative of the outgroup. Branches leading to genes from the different phyla are coloured according to the simplified phylogeny of land plants that is shown in the top left corner. The phylogenetic position of some known type II MIKC^c^ MADS-box genes^111^ representative of previously described clades of MADS-box genes are indicated on the right together with the species and phylum in which these genes have been identified. The four clades of MIKC^c^ MADS-box genes that trace back to the most recent common ancestor of Euphyllophytes are shaded in grey.

Whereas none of our focal gene families exhibited significant expansion in ferns, significantly more MIKC-type MADS-box genes—involved in specification of ovule and flower development in seed plants^52^—were observed in leptosporangiate ferns relative to all other green plant lineages, other than seed plants (Extended Data Fig. 1). The ancestral number of MIKC-type MADS-box genes for ferns and seed plants was 4 or 5, and gene numbers increased independently within leptosporangiate ferns and seed plants (Extended Data Figs. 1, 2).

### Seed plants

A WGD in the ancestry of all extant seed plants has been inferred previously^18,53^ but remains contested^54^. Gene-tree^18^ analyses revealed significantly more gene duplications on the branch leading to extant seed plants than expected from background gene birth and death rates (analyses D1 (*P* < 2.0 × 10^−18^) and D2 (*P* < 8.9 × 10^−16^) in Supplementary Table 5). Numerous gene-family expansions were also associated with the origin of seed plants, and only one contraction was detected among the gene families analysed (Fig. 5b). Type II MIKC-type MADS-box genes exhibited a nearly twofold expansion independent of their expansion in ferns (Extended Data Figs. 1, 2).

Extant gymnosperms (approximately 1,000 species) are sister to flowering plants, and all of our analyses recovered Cycadales and *Ginkgo* (Fig. 1l) as a sister clade to the remaining gymnosperms (Fig. 3c). The placement of Gnetales conflicts strongly among the ASTRAL, supermatrix and plastome-based trees. Plastid data strongly support the ‘Gnecup’ hypothesis, with Gnetales as sister to a clade comprising Araucariales and Cupressales^47^, whereas the supermatrix analysis of nuclear genes supports a ‘Gnepine’ hypothesis with Gnetales as sister to Pinales^55,56^. ASTRAL analyses strongly support the ‘Gnetifer’ hypothesis, with conifers (Araucariales, Cupressales and Pinales) sister to Gnetales^57^. The short internal branches in the ASTRAL tree suggest rapid diversification (Fig. 2). However, the uneven frequencies of gene-tree quartets—which support the alternative Gnecup and Gnepine hypotheses—suggest that gene-tree estimation biases^58^ associated with increased substitution rates in Gnetales^59^ or gene flow are possible sources of gene-tree discordance^8^. Previously inferred WGDs in ancestors of *Welwitschia*, Pinaceae and Cupressales^18^ are supported, as is a new inference of WGD in the ancestry of Podocarpaceae (Fig. 4 and Supplementary Tables 2, 5, 6).

Angiosperms are by far the largest clade of green plants (more than 370,000 species^2^) and are marked by multiple key innovations, including the carpel, double fertilization, endosperm, and for most angiosperms, vessel elements. Both nuclear and plastid phylogenomic analyses agree with previous studies^39^ in providing strong support for angiosperm monophyly and in placements of Amborellales, Nymphaeales and Austrobaileyales as successive sisters to all other angiosperms (Figs. 2, 3). Chloranthales and magnoliids comprise a clade in the ASTRAL and supermatrix analyses, but were resolved with poor support as successive sister lineages to all other Mesangiospermae (monocots, *Ceratophyllum* and eudicots) in the plastome-based tree. Whereas *Ceratophyllum* is sister to eudicots in the ASTRAL and plastome trees, it is poorly supported as sister to monocots in the supermatrix tree (Supplementary Figs. 1–3). All analyses suggest short time intervals between branching of the monocots, Magnoliidae, Chloranthales, Ceratophyllales and eudicot lineages in early mesangiosperm history (Fig. 2 and Supplementary Figs. 1–3).

Pentapetalae (70% of all angiosperms) are marked by the evolution of the pentamerous flower. Substantial gene-tree discordance was observed for relationships among core rosids, Saxifragales, Vitales, *Dillenia*, Santalales, Berberidopsidales, Caryophyllales, asterids and Gunnerales (the sister group of Pentapetalae; Fig. 3i). Short internal branches and poor support in the ASTRAL tree at the base of the core eudicots (Figs. 2, 3i) indicate rapid diversification following two rounds of WGD that resulted in palaeohexaploidy preceding the origin of the clade^60,61^ (Supplementary Fig. 8). The supermatrix and plastid trees conflict with the poorly supported ASTRAL branching order (Fig. 3i). With the exception of the Berberidopsidales and core asterid clade, we were not able to reject the possibility of polytomies at the evaluated nodes in ASTRAL analyses (Fig. 3i).

Genomic and phylogenomic analyses have identified numerous WGDs throughout angiosperm history^62,63^. We found evidence that extant flowering plants descend from a polyploid common ancestor^19,53^. Gene-tree analyses detected a significantly larger-than-background number of gene duplications on the branch leading to the last common ancestor of extant angiosperms after divergence from the extant gymnosperm clade (analyses E1 (*P* < 1.8 × 10^−41^) and E2 (1.4 × 10^−24^) in Supplementary Table 5). Furthermore, the numbers of inferred duplications on the stem branch of angiosperms were consistent with expectations for WGD (analyses E1 and E2 in Supplementary Table 6). We inferred over 180 WGDs within flowering plants, including 132 in eudicots and 35 in monocots (Supplementary Table 2).

The origin of the angiosperms was preceded by three focal gene-family contractions and no expansions (Fig. 5b), consistent with the hypothesis that the innovations in angiosperms may have involved the functional co-option of genes that were duplicated earlier in the evolution of seed plants^19^. We find that orthologues of some floral homeotic MADS-box genes originated in the stem group of extant seed plants approximately 300 million years ago (Extended Data Fig. 2), supporting the hypothesis that the origin of the angiosperm flower involved recruitment of developmental regulators that already existed in their seed plant ancestors^19,64^.

## Synthesis

These analyses establish a foundation for advancing our understanding of the overall phylogenetic framework of green plants and the genetic changes that were responsible for the characteristic traits associated with major evolutionary transitions in Viridiplantae. Portions of the species tree reported here remain unresolved. Phylogenetic analyses of genes extracted from a broad sampling of whole-genome sequences may improve gene family circumscriptions and resolve the species tree further. Expanded genome sequencing may also help to accurately account for interspecific gene flow, and orthology in the face of gene duplications and losses. However, for some nodes in the species tree, extensive discordance among inferred gene histories suggests that rapid diversification may not always conform to strict bifurcation of ancestral species into two descendent species.

Gene and genome duplications have long been considered a source of evolutionary novelty^11,12^, producing an expanded molecular repertoire for adaptive evolution of key pathways and shifts in plant development and ecology. However, the direct connections between key innovations and specific gene duplications are rarely known, due in part to lag times between duplications and such inovations^25–27^. Phylogenetically informed experimental investigations of changes in gene content and function will improve our understanding of the roles of gene and genome duplications in the evolution of key innovations. Such efforts are underway, drawing on an expanding number of experimental model species distributed across the green plant tree of life^65^.

## Methods

### Data reporting

No statistical methods were used to predetermine sample size. The experiments were not randomized, although simulations included in the genome duplication analyses did include drawing from random distributions. The investigators were not blinded to allocation during experiments and outcome assessment.

### Transcriptome sequencing

RNA was isolated from young vegetative tissue from all of the species that were included in our phylogenomic analyses as described elsewhere^39,67,68^. Reproductive tissues were also included for some species (Supplementary Table 1). Transcript assembly, contaminant identification and gene-family circumscription were also performed as described previously^39^ and are described in more detail in the Supplementary Methods.

### Phylogeny reconstruction

Analyses were performed on single-copy gene trees using ASTRAL to account for variation among gene trees owing to incomplete lineage sorting^15,69^. ASTRAL analyses were performed on gene trees estimated from unbinned amino acid alignments, first and second codons, statistically binned supergenes with unweighted bins^70,71^ and filtered taxon sets (excluding ‘rogue’ taxa as described below), with filtering of gene-tree bootstrap support thresholds of up to 33% to see whether the effects of gene-tree estimation error could be reduced (Supplementary Fig. 6). Binning left the majority of genes in singleton bins and had minimal effects on the overall species tree. Unless otherwise specified, we use ‘ASTRAL topology’ to refer to the tree inferred from 410 unbinned amino acid alignments in which branches with 33% or less support are contracted. In addition, supermatrix analyses were performed on concatenated nuclear gene alignments and concatenated plastid gene alignments compiled using previously described methods^72^. All scripts used to perform analyses on the nuclear gene data are available at 10.5281/zenodo.3255100.

#### Multiple sequence alignment and data filtering

We built a multiple sequence alignment based on predicted amino acid sequences of each gene and forced DNA sequences to conform to the amino acid alignment. We first divided sequences in each gene into two subsets, full-length and abnormal sequences, and then used PASTA^73^ with default settings to align full-length sequences and UPP^74^ to add abnormal sequences to the full-length alignment. We designated as abnormal any sequence that was 66% shorter or 66% longer than the median length of the full-length gene sequences. Once UPP alignments were obtained, we removed from them all unaligned (that is, insertion) sites. DNA alignments were then derived from amino acid sequence alignments (FAA2FNA) and third codon positions were removed owing to extreme among-species variation in GC content (Supplementary Fig. 7). To reduce running time, we then masked all sites from the alignment that contained more than 90% gaps. Finally, because the inclusion of fragmentary data in gene-tree estimation can be problematic^75^, we removed any sequence that had a gap for at least 67% of the sites in the site-filtered alignment (the 67% threshold was chosen based on simulation results^75^). Gene sequence occupancy for 410 single-copy genes in the 1,178 accessions used in our analyses is displayed as a frequency histogram (Supplementary Fig. 4) and a heat map (Supplementary Fig. 5).

In addition to filtering gappy sites and fragmentary sequences, we identified and removed sequences that were placed on extremely long branches on their respective gene trees. To identify these, we used the initial alignments to build gene trees (see below). We then rooted each gene tree by finding the bipartition that separated the largest exclusive group of outgroup or red algae taxa. If red algae were entirely missing for the gene, we used Glaucophyta, Prasinococcales, prasinophytes, *Volvox carteri*, *Chlamydomonas reinhardtii* or *Klebsormidium nitens*. We then removed any sequences that had a root-to-tip distance that was four standard deviations longer than the median root-to-tip distance in each gene tree. Once these sequences on long branches were removed, alignments were re-estimated using the same approach described above, and new gene trees were estimated.

#### Gene-tree estimation

To estimate gene trees, we used RAxML v.8.1.17^76^, with one starting tree for building initial trees (used for long-branch filtering) and 10 different starting trees for final gene trees. Support was assessed with 100 replicates of bootstrapping. For DNA analyses, the GTR substitution model and the GAMMA-distributed site rates were used. For amino acid sequences, we used a Perl script adapted from the RAxML website to search among 16 different substitution models on a fixed starting tree per gene and chose the model with the highest likelihood (JTT, JTTF or JTTDCMUT were selected for 349 out of 410 genes). For amino acid trees, we also used the GAMMA-distributed site rates.

#### Species tree estimation

We used ASTRAL-II^15^ v.5.0.3 to estimate the species tree on the basis of all 410 genes; using 384 genes that each included at least half of the species changed only 3 low-support branches. We used multi-locus bootstrapping^77,78^ and the built-in local posterior probabilities of ASTRAL to estimate branch support^69^ and to test for polytomies^79^, drawn on species trees estimated based on the maximum-likelihood gene trees. We also used the built-in functionality of ASTRAL (version 4.11.2) to compute the percentage of gene trees that agreed with each branch in the species tree, by finding the average number of gene-tree quartets defined around the branch (choosing one taxon from each side) that were congruent with the species tree and used DiscoVista^80^ to visualize them (Fig. 4). Median representation of each species across the 410 single-copy gene trees was 82.4% with 88.2% and 67.1% of species having assemblies for at least 50% or 75% of the 410 single-copy genes, respectively. A large body of work on phylogenetic methodologies has established that gene and species tree estimation can be robust to missing data, particularly with dense taxon sampling^75,81,82^. Recent papers have even established statistical consistency under missing data^83^. Similar evidence of robustness also exists in the context of concatenated analyses^84–86^.

All supermatrix analyses are based on the filtered amino acid and first and second codon position alignments that included at least half of the species for 384 genes. The (1) unfiltered supermatrices used the gene alignments as is; the (2) eudicot supermatrices retained only eudicot species in the supermatrix; and the (3) supermatrices with eight ‘rogue’ taxa removed (*Dillenia indica*, *Tetrastigma obtectum*, *Tetrastigma voinierianum*, *Vitis vinifera*, *Cissus quadrangularis*, ‘*Spirotaenia*’ sp., *Ceratophyllum demersum* and *Prasinococcus capsulatus*) that varied in placement among our full ASTRAL, supermatrix and plastid genome analyses. Well-supported branching orders were stable among analyses (Supplementary Fig. 6).

Maximum-likelihood supermatrix analyses were performed using ExaML v.3.0.14^87^. Similar to the gene-tree analyses, the GAMMA model of rate heterogeneity across sites was used for all maximum-likelihood supermatrix analyses. To better handle model heterogeneity across genes, we divided the supermatrix into partitions. For the amino acid alignments, the protein model selected for each gene family in the gene-tree estimation process was used to group genes into partitions, creating one partition per substitution model. For the nucleotide alignments, we estimated the GTR transition rate parameters and the alpha shape parameter for each codon position (first and second positions) of each alignment using RAxML v.8.1.21^76^. We then projected the maximum-likelihood parameter values for each gene into a two-dimensional plane using principal component analysis^88^. We performed *k*-means clustering^89^ in R^90^ to group the codon positions into partitions, selecting *k* = 8, which accounted for 80% of the variation. Trees derived from nucleotide alignments can be found at 10.5281/zenodo.3255100).

To examine the influence of the starting tree on the likelihood of the final tree, we performed preliminary analyses on an earlier version of our supermatrices. We generated nine different maximum-parsimony starting trees using RAxML v.8.1.21 and one maximum-likelihood starting tree using FastTree-2 v.2.1.5^91^. We then ran ExaML on each of the starting trees, noting the final maximum-likelihood score. We found that in all cases, the ExaML maximum-likelihood tree using the FastTree-2 maximum-likelihood starting tree had a better maximum-likelihood score than any of the ExaML maximum-likelihood trees using maximum-parsimony starting trees. Thus, for all of the supermatrix analyses, we used FastTree-2 to generate our initial starting tree. Support was inferred for the branches of the final tree from 100 bootstrap replicates.

Outgroup taxa from outside Archaeplastida were used to root all species trees estimated using nuclear genes (all ASTRAL and supermatrix analyses). The plastome supermatrix tree for Viridiplantae was rooted using Rhodophyta as outgroup.

### Inferring and placing WGDs

#### DupPipe analyses of WGDs from transcriptomes of single species

For each transcriptome, we used the DupPipe pipeline to construct gene families and estimate the age distribution of gene duplications^16,17^. We translated DNA sequences and identified reading frames by comparing the Genewise^92^ alignment to the best-hit protein from a collection of proteins from 25 plant genomes from Phytozome^93^. For all DupPipe runs, we used protein-guided DNA alignments to align our nucleic acid sequences while maintaining the reading frame. We estimated synonymous divergence (*K*_s_) using PAML with the F3X4 model^94^ for each node in the gene-family phylogenies. We identified peaks of gene duplication as evidence of ancient WGDs in histograms of the age distribution of gene duplications (*K*_s_ plots). We identified species with potential WGDs by comparing their paralogue age distribution to a simulated null using a Kolmogorov–Smirnov goodness of fit test^95^. We then used mixture modelling and manual curation to identify significant peaks consistent with a potential WGD and to estimate their median paralogue *K*_s_ values. Significant peaks were identified using a likelihood ratio test in the boot.comp function of the package mixtools in R^96^.

#### Estimating orthologous divergence

To place putative WGDs in relation to lineage divergence, we estimated the synonymous divergence of orthologues among pairs of species that may share a WGD based on their phylogenetic position and evidence from the within-species *K*_s_ plots. We used the RBH Orthologue pipeline^17^ to estimate the mean and median synonymous divergence of orthologues and compared those to the synonymous divergence of inferred paleopolyploid peaks. We identified orthologues as reciprocal best blast hits in pairs of transcriptomes. Using protein-guided DNA alignments, we estimated the pairwise synonymous divergence for each pair of orthologues using PAML with the F3X4 model^94^. WGDs were interpreted to have occurred after lineage divergence if the median synonymous divergence of WGD paralogues was younger than the median synonymous divergence of orthologues. Similarly, if the synonymous divergence of WGD paralogues was older than that orthologue synonymous divergence, then we interpreted those WGDs as shared.

#### MAPS analyses of WGDs from transcriptomes of multiple species

To infer and locate putative WGDs in our datasets, we used a gene-tree sorting and counting algorithm, the multi-taxon paleopolyploidy search (MAPS) tool^18^. For each MAPS analysis, we selected at least two species that potentially share a WGD in their ancestry as well as representative species from lineages that may phylogenetically bracket the WGD. MAPS uses this given species tree to filter collections of nuclear gene trees for subtrees consistent with relationships at each node in the species tree. Using this filtered set of subtrees, MAPS identifies and records nodes with a gene duplication shared by descendant taxa. To infer and locate a potential WGD, we compared the number of duplications observed at each node to a null simulation of background gene birth and death rates^97,98^. A Fisher’s exact test, implemented in R^90^, was used to identify locations with significant increases in gene duplication compared with a null simulation (Supplementary Table 5). Locations with significantly more duplications than expected were then compared to a simulated WGD at this location. If the observed duplications were consistent with this simulated WGD using Fisher’s exact test, we identified the location as a WGD if it was consistent with inferences from *K*_s_ plots and orthologue divergence data. In some cases, MAPS inferred significant duplications without apparent signatures in *K*_s_ plots or previously published research. In these cases, we recognized the event as a significant burst of gene duplication.

Each MAPS analysis was designed to place focal WGDs near the centre of a species tree to minimize errors in WGD inference. Errors in transcriptome or genome assembly, gene-family clustering and the construction of gene-family phylogenies can result in topological errors in gene trees^99^. Previous studies have suggested that errors in gene trees can lead to biased placements of duplicates towards the root of the tree and losses towards the tips of the tree^100^. For this reason, we aimed to put focal nodes for a particular MAPS analysis test in the middle of the phylogeny. To further decrease potential error in our inferences of gene duplications, we required at least 45% of the ingroup taxa to be present in all subtrees analysed by MAPS^97^. If this minimum requirement of ingroup taxa numbers is not met, the gene subtree will be filtered out and excluded from our analysis. Increasing taxon occupancy leads to a more accurate inference of duplications and reduces some of the biases in mapping duplications onto a species tree^100,101^. To maintain sufficient gene-tree numbers for each MAPS analysis, we used collections of gene-family phylogenies for six to eight taxa to infer ancient WGDs.

For each MAPS analysis, the transcriptomes were translated into amino acid sequences using the TransPipe pipeline^17^. Using these translations, we performed reciprocal protein BLAST (BLASTp) searches among datasets for the MAPS analysis using a cut-off of *E* = 1 × 10^−5^. We clustered gene families from these BLAST results using OrthoFinder under the default parameters^102^. Using a custom Perl script (https://bitbucket.org/barkerlab/maps), we filtered for gene families that contained at least one gene copy from each taxon in a given MAPS analysis and discarded the remaining OrthoFinder clusters. We used PASTA^73^ for automatic alignment and phylogeny reconstruction of gene families. For each gene-family phylogeny, we ran PASTA until we reached three iterations without an improvement in likelihood score using a centroid breaking strategy. Within each iteration of PASTA, we constructed subset alignments using MAFFT^103^, used Muscle^104^ for merging these subset alignments and RAxML^76^ for tree estimation. The parameters for each software package were the default options for PASTA (https://bitbucket.org/barkerlab/1kp). We used the best-scoring PASTA tree for each multi-species nuclear gene family to collectively estimate the numbers of shared gene duplications on each branch of the given species.

To generate null simulations, we first estimated the mean background gene duplication rate (*λ*) and gene loss rate (*μ*) with WGDgc^98^ (Supplementary Tables 5, 11). Gene count data were obtained from OrthoFinder^102^ clusters associated with each species tree (Supplementary Table 5). *λ* and *μ* were estimated using only gene clusters that spanned the root of their respective species trees, which has been shown to reduce biases in the maximum-likelihood estimates^98^ of *λ* and *μ*. We chose a maximum gene-family size of 100 for parameter estimation, which was necessary to provide an upper bound for numerical integration of node states^98^. We provided a prior probability distribution on the number of genes at the root of each species tree, such that ancestral gene-family sizes followed a shifted geometric distribution with mean equal to the average number of genes per gene family across species (Supplementary Table 5).

Gene trees were then simulated within each MAPS species trees using the GuestTreeGen program from GenPhyloData^105^. For each species tree, we simulated 3,000 gene trees with at least one tip per species: 1,000 gene trees at the *λ* and *μ* maximum-likelihood estimates, 1,000 gene trees at half the estimated *λ* and *μ*, and 1,000 trees at three times *λ* and *μ*. For all simulations, we applied the same empirical prior used for estimation of *λ* and *μ*. We then randomly resampled 1,000 trees without replacement from the total pool of gene trees 100 times to provide a measure of uncertainty on the percentage of subtrees at each node. For positive simulations of WGDs, we simulated gene trees using the same approach used to generate null distributions (Supplementary Table 5) but incorporated a WGD at the test branch. Previous empirical estimates of paralogues retained following a plant WGD are 10% on average^106^. To be conservative for inferring WGDs in our MAPS analyses, we allowed at least 20% of the genes to be retained following the simulated WGD to account for biased gene retention and loss. For WGDs that might have a lower gene retention rate, we used an additional simulation using 15% gene retention (Supplementary Table 6).

### Gene-family evolution

#### Transcriptome-based gene-family size estimation

To robustly estimate gene-family sizes from transcriptomic data, we needed to overcome three major challenges: (1) the fragmentation of transcript sequences; (2) the absence of low-abundance transcripts; and (3) the over-prediction of gene-family sizes due to assembly duplications and biological isoforms. We dealt with these challenges as follows.

#### Fragmentation of data

The multiple sequence alignments used to construct the domain-specific profile hidden Markov models (HMMs) ranged from 23 to 463 amino acids in length; 78% of these alignments were shorter than 120 amino acids, and 84.6% of the assembled and translated transcripts were longer than 120 amino acids. By mainly characterizing gene families using single domains (Supplementary Table 9), we limited the effect of the fragmentation of transcripts from the assembly of short read data. HMMs used for gene-family classification and decision rules obtained from either published work^107^ or gene-family experts are given in Supplementary Table 9; 12 out of 23 gene families were classified by a single ‘should’ rule, 2 out of 23 were defined by a XOR ‘should’ rule, which also classifies a sequence by the presence of a single domain, 8 out of 23 gene families were classified by a more complex rule set including ‘should not’ rules. The only gene family for which multiple domains needed to be present was the PLS subfamily of the PPR gene family.

#### Loss of low abundance transcripts

To account for possible bias in the sampling of the gene space, all species that showed low levels of transcriptome completeness were removed. The lowest value of transcriptome completeness obtained from 30 annotated plant genomes was used as the lower exclusion limit. We removed all samples in which more than 42.5% of BUSCO^31^ sequences were missing using default settings and the eukaryotic dataset as the query database.

#### Gene-family over-prediction

We clustered assembled protein sequences by sequence similarity and merged sequences that showed at least 99% identity. To check for the possibility of merging sequences that should be counted separately, different identity cut-offs were compared between the 1KP datasets and 32 annotated plant genomes.

**Extended Data Fig. 3:**
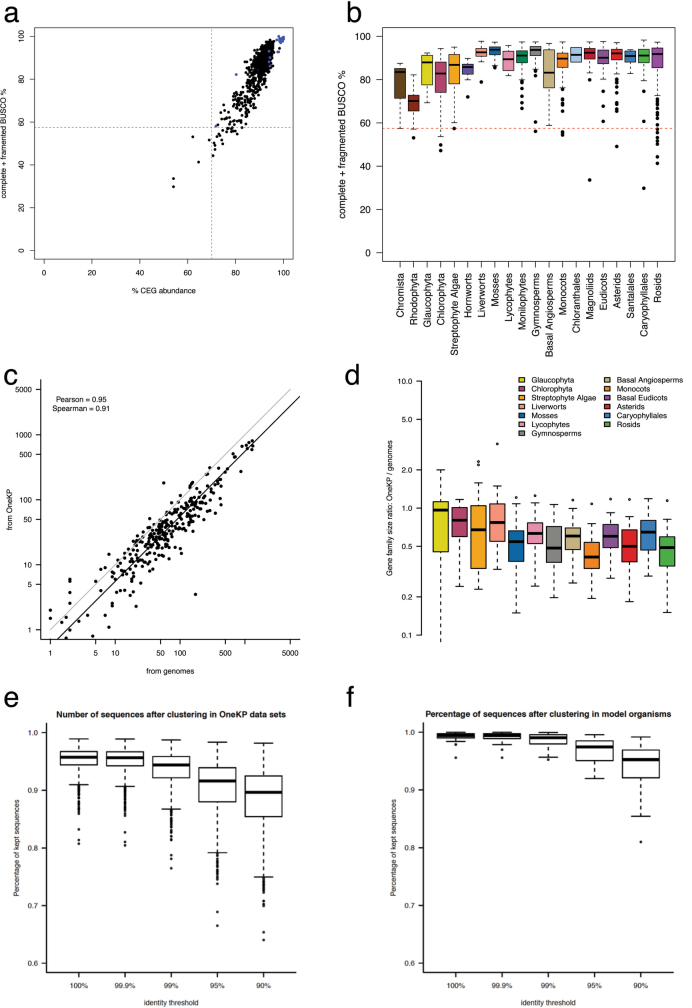
Assessments of transcriptome assembly gene-family representation relative to gene-family members identified in annotated genomes. **a**, BUSCO versus CEGMA (CEG) gene occupancy for each sample. BUSCO transcriptome completeness is given as ‘complete plus fragmented’ BUSCO percentage using the eukaryota_odb9 database. CEGMA transcriptome completeness is given as conditional reciprocal best BLAST hits (see Supplementary Methods). Dotted line represents 57.5% (BUSCO) and 70% (CEGMA) gene occupancy threshold. Black dots represent 1KP samples (*n* = 1,020) and blue dots annotated plant genomes (*n* = 30). **b**, BUSCO gene occupancy for each major clade. Boxes represent lower and upper quartiles; the black bold line represents the median and whiskers extend to the most-extreme data points. Sample sizes: Chromista, *n* = 23; Rhodophyta, *n* = 18; Glaucophyta, *n* = 2; Chlorophyta, *n* = 94; streptophyte algae, *n* = 42; hornworts, *n* = 7; liverworts, *n* = 18; mosses, *n* = 38; lycophytes, *n* = 16; monilophytes, *n* = 59; gymnosperms, *n* = 76; ANA grade, *n* = 6; monocots, *n* = 96; Chloranthales, *n* = 1; magnoliids, *n* = 22; CRPT grade, *n* = 29; asterids, *n* = 205; Caryophyllales, *n* = 48; rosids, *n* = 176; Saxifragales, *n* = 23; Santalales, *n* = 6. Dotted line represents 57.5% (BUSCO) gene occupancy threshold. **c**, Scatterplot of gene-family sizes in transcriptomes versus genomes on a logarithmic scale. The grey line indicates *x* = *y*, the black line indicates a linear regression fitted to the data (*n* = 299; 23 gene families in 13 species groups). Pearson and Spearman correlation coefficients (*n* = 299) are indicated. **d**, Box plot of transcriptome:genome ratios of gene-family sizes for each species group. Boxes indicate upper and lower quartiles with median; whiskers extend to data points no more than 1.5× the interquartile range (*n* = 23) with outliers plotted as individual data points. **e**, **f**, Number of remaining sequences after filtering with cd-hit and a threshold of 100%, 99.9%, 99%, 95% or 90% in transcriptome sequences and reference genomes (Supplementary Table 8). Boxes indicate upper and lower quartiles with median; whiskers extend to data points no more than 1.5× the interquartile range (**e**, *n* = 1,451; **f**, *n* = 32) with outliers plotted as individual data points.

Extended Data Figure 3c, d shows the average gene-family sizes for 23 gene families and 13 clades obtained from 1KP samples and 32 annotated plant genomes. These gene-family sizes show a high Pearson correlation (*r* = 0.95) between 1KP samples and plant genomes, and therefore a linear relationship between the two approaches is indicated. The results from the 1KP dataset are on average smaller by a factor of 2.3. Although this is a clear underestimate, the scale factor by which the estimate is too small is relatively consistent, especially as the gene-family sizes increase.

#### Sequence clustering

We used cdhit v.4.5.7^108,109^ to reduce the number of protein sequence duplications in the dataset. We assessed 100%, 99.5%, 99%, 95% and 90% sequence identity thresholds. The percentage of remaining sequences for the 1KP samples and 32 reference genomes is displayed in Extended Data Fig. 3f. We chose 99% sequence identity as the value to use for this study.

#### Estimation of gene-family size

Gene-family experts provided the knowledge to classify protein sequences as members of gene families with profile HMMs. In total, 46 HMMs representing 23 large gene families^30^ were used to estimate gene-family sizes in the analysed species. Classification rules and HMMs for 14 gene families that have been published previously^107^ were converted to HMMER3 format and used in this study. Gene-family classification rules and HMMs for the remaining nine families can be found in Supplementary Table 8. HMMs were taken from the Pfam database (accessed 12 May 2016) or were provided by gene-family experts (Supplementary Table 8). HMMER^110^ (v.3.1b2) was used to scan for matches in the filtered 1KP dataset. Where available, gathering thresholds were used; otherwise an *E*-value cut-off was applied to indicate domain presence. If the *E* value is not noted in Supplementary Table 9, the default *E* value of 10 was applied. The results on the species level are listed in Supplementary Table 10s.

#### Statistical test for expansions and contractions

To assess whether a gene family expanded or contracted in a lineage, we compared a weighted average of gene numbers in adjacent clades and grades (Fig. 4). We also checked for expansions and contractions within clades but did not find any statistically significant shifts. The counts of gene-family members from two clades or grades were compared with a Kolmogorov–Smirnov test with a *P*-value threshold of 1 × 10^−6^ in R^90^. The tests conducted in this study are listed in Supplementary Table 7. Fold changes were computed using the trimmed arithmetic mean in which the top and bottom 5% of the data were discarded. Only expansions larger than 1.5 fold (or contractions smaller than 2/3) are reported.

### Reporting summary

Further information on research design is available in the Nature Research Reporting Summary linked to this paper.

## Online content

Any methods, additional references, Nature Research reporting summaries, source data, extended data, supplementary information, acknowledgements, peer review information; details of author contributions and competing interests; and statements of data and code availability are available at 10.1038/s41586-019-1693-2

## Supplementary information

Supplementary Information This file contains the Supplementary Methods which include detailed descriptions of methods for transcriptome data generation and assessment, phylogenetic analyses, inference of whole genome duplications (WGDs) and gene family expansions. Supplementary Results are also included with additional details for results of phylogenetic and WGD analyses. Supplementary Figures 1-8 are included at the end of the file.

Reporting Summary

Supplementary Table 1 **Sample list** including 1KP indices, species names and lineage taxonomy, RNA seq data volumes, assembly assessments (BUSCO and TransRate scores) and SRA submission indices.

Supplementary Table 2 **Summary of 244 inferred ancient WGDs from the 1KP analyses**. WGD ID codes are organized in alphabetical order. The phylogenetic placement of each inferred ancient WGD is provided in Extended Data Fig. 4. An * indicates presence and absence of WGDs from cited syntenic analyses of whole genome data.

Supplementary Table 3 **Summary of Ks distributions of duplicate gene pairs for each species**. Species name and median Ks for each histogram of the age distribution of gene duplications (Ks plots available at https://bitbucket.org/barkerlab/1kp), and p-values of the two-sided K-S goodness of fit test is provided for each taxon. The median Ks value, the number of WGD peak inferred from Ks plots range from Ks 0 to 2 and 0 to 5 using mixture models from mixtools R package are reported. The sample size used to estimate the median Ks value, n=number of gene duplications, was calculated from the total number of gene duplications for each species multiplied by the percent contribution of a particular peak by mixtools. The number of inferred WGD in the ancestry of each species is also reported.

Supplementary Table 4 **Summary of the synonymous ortholog divergence analyses**. The mean, median, and standard deviation of synonymous ortholog divergence (Ks) for each inferred ancient WGD is reported. These statistics are based on the number of ortholog pairs identified for each species pair comparison. The sampling information with taxon code and WGD code is also reported.

Supplementary Table 5 **Summary statistics and null simulations (no WGDs) for 72 Multi-tAxon Paleopolyploidy Search (MAPS) analyses**. For each node of 72 MAPS analyses percentage of subtrees with shared inferred gene duplications and numbers of gene duplications in simulations without WGDs are reported along with the p-value for a one-sided Fisher’s exact test used to detect nodes with a significantly higher proportion of inferred gene duplications compared to the null distribution. An * indicates a significant node. Results are organized in tabs for each major green plant taxon.

Supplementary Table 6 **Summary statistics and power simulations with WGDs for 72 MAPS analyses**. As with Supplementary Table 5, for each lineage in each MAPS tree percentages of subtrees with shared inferred gene duplications is reported along with expectations bases on simulations with 20% (or 15% for six selected analyses) paralog retention following WGDs. Tables contains the p-value for a one-sided Fisher’s exact test used to detect nodes with a significantly lower proportion of mapping subtrees compared to our simulation. Results are organized in tabs for each major green plant taxon.

Supplementary Table 7 **Gene family expansion counts for each taxon and gene family shown in** Fig. 5.

Supplementary Table 8 **Reference genomes used in phylogenetic analyses, Orthofinder gene family circumscription, and gene family size analyses.**

Supplementary Table 9 **Description of gene family HMMs and gene family experts.**

Supplementary Table 10 **Gene family HMM search statistics for each of the samples listed in Supplementary Table 1.**

Supplementary Table 11 **Rates of gene duplication (λ) and gene loss (μ) used in null and positive simulations (Supplementary Tables 5-6)**. Rates of gene duplication (λ) and gene loss (μ) were estimated using gene counts from OrthoFinder clusters associated with each MAPS analysis. Values correspond to global Maximum Likelihood Estimates (MLEs) and mean rates for simulations. The prior mean is the mean of the geometric probability distribution applied to the root of each species tree for optimizing MLEs of λ and μ as well as simulating gene trees with and without WGDs.

## Data Availability

All raw sequence reads have been posted in the NCBI SRA database under BioProject accession PRJEB4922. SRA entries for each assembly are listed in Supplementary Table 1. All sequence, gene tree and species tree data can be accessed through CyVerse Data Commons at 10.25739/8m7t-4e85. In addition, gene-family nucleotide and amino acid FASTA files can also be found at http://jlmwiki.plantbio.uga.edu/onekp/v2/; multiple sequence alignments, gene trees and species trees for single-copy nuclear genes included in phylogenomic analyses are also at 10.5281/zenodo.3255100; *K*_s_ plots, alignments and trees used for WGD analyses can be found at https://bitbucket.org/barkerlab/1kp; and data used for gene-family expansion analyses can be found at https://github.com/GrosseLab/OneKP-gene-family-evo.

## Extended data figures and tables

A list of participants and their affiliations appears in the online version of the paper.

## Code availability

Scripts used for phylogenomic species tree analyses are available at 10.5281/zenodo.3255100. Scripts used for MAPS analyses of WGDs are available at https://bitbucket.org/barkerlab/maps and scripts used for gene-family expansion analyses are available at https://github.com/GrosseLab/OneKP-gene-family-evo. All script files are also accessible through CyVerse Data Commons at 10.25739/8m7t-4e85.
