# Supplementary material for: One thousand plant transcriptomes and the phylogenomics of green plants
Source: Nature. 2019 Oct 23;574(7780):679–85. doi: 10.1038/s41586-019-1693-2 (PMC6872490; doi:10.1038/s41586-019-1693-2)
Supplement: Supplementary file 1 — This file contains the Supplementary Methods which include detailed descriptions of methods for transcriptome data generation and assessment, phylogenetic analyses, inference of whole genome duplications (WGDs) and gene family expansions. Supplementary Results are also included with additional details for results of phylogenetic and WGD analyses. Supplementary Figures 1-8 are included at the end of the file. [file 41586_2019_1693_MOESM1_ESM.pdf]

nature

https://doi.org/10.1038/s41586-019-1693-2

Supplementary information

One thousand plant transcriptomes and the phylogenomics of green plants

In the format provided by the authors and unedited

One Thousand Plant Transcriptomes Initiative

Supplemental Methods

Transcriptome data generation

Sample acquisition: The 1000 plants initiative (1KP or onekp) analyzed 1342 RNA seq libraries from 1147 species including 1112 green plant species representing all of the major taxa within the Viridiplantae, including angiosperms, conifers, ferns, mosses, streptophytic algae and chlorophyte green algae (Supplementary Table 1). Because of the diversity of species, no one source could be used. Samples were provided by a global network of more than a hundred tissue and RNA providers (see author contributions) who obtained materials from a variety of sources, including field collection of wild plants, botanical gardens, greenhouses, laboratory specimens, and culture collections. Typically, samples were fast growing tissues such as young leaves or shoots, because these are expected to be largely comprised of live cells and therefore rich in RNA. However, some RNAs were derived from a mix of vegetative and reproductive tissues and we did not attempt to define specific standards on growth conditions, time of collection, or age of tissue.

Vouchering system: The majority of the samples have corresponding vouchers deposited at various institutions, as convenient for the sample providers (http://www.onekp.com/samples/list.php). For the algal species, material from culture collections was sourced and the relevant collection identifiers are reported. Species names were validated with the Taxonomic Name Resolution Service[1](http://tnrs.iplantcollaborative.org) or checked against other plant name databases such as Tropicos (http://www.tropicos.org). Numerous minor issues in species names were resolved in this way.

RNA extraction: Because RNA is expected to degrade rapidly after a sample is taken, RNA extractions were either performed immediately after tissue collection, or tissue was flash frozen in liquid nitrogen and stored at or below -80˚C until extraction. Given the biochemical diversity of our samples, no one RNA extraction protocol was appropriate for every instance. Most of the samples were extracted with common extraction protocols such as CTAB, TRIzol, hot acid-phenol, or with commercial products such as Invitrogen Purelink or Qiagen RNeasy kits and variants thereof. For complete details of specific protocols please see Appendix S1 of Johnson et al.[2] and Jordon-Thaden et al.[3]. Depending on the sample, the RNA extraction might have been done at the sequencing lab or by one of the sample providers. RNA was either shipped on dry ice or (for 36% of cases) at ambient temperatures in GenTegra.

Sequencing at BGI: Before library construction, RNA quality was assessed by a Bioanalyzer spectrogram (typically requiring RIN scores of greater than 6) and basic photometery for obvious low-quality outliers. BGI used either in house protocols or TruSeq chemistry (low-mass cases) to create sequencing libraries of ~200 bp insert sizes. Initially, paired-end sequencing was done with Illumina GAII machines, but later samples were run on the HiSeq platform. Associated with this change was a shift from a ~72 bp read length to a 90 bp read length at each end. Libraries were barcoded and multiplexed on the sequencer lanes to a target sequencing depth of at least 2 Gbp of Q20 data per sample.

Percentile	Dataset Size
5th	1.3 Gbp
25th	1.9 Gbp
50th	2.2 Gbp
75th	2.5 Gbp
95th	3.0 Gbp

After removal of read-pairs with various low-quality indications, and demultiplexing, the sequence was provided to the rest of the consortium for further analysis.

De novo assembly: Quality filtered reads were assembled *de novo* using the SOAPdenovo-Trans assembler (version of 2012-04-05)[4]. No additional pre-processing of the data was performed. The assembly was done by running both the main assembler and the GapCloser post-processor programs. Options were the program defaults, except that the *k*-mer length was increased to 25 bp.

Assembly qualities: We assessed the quality of each assembled scaffold using TransRate[5] which maps reads to assemblies to assign a quality score to each scaffold as an assessment of accuracy, fragmentation and completeness[5]. Overall TransRate scores are the geometric mean of all contig scores multiplied by the proportion of reads mapping to the assembly. Scores ranged between 0.012 and 0.393 (median = 0.103; Supplementary Table 1), in line with the range of scores reported for NCBI SRA assemblies assessed in the TransRate publication (range 0-0.523, median = 0.217)[5]. The representation of conserved genes was assesses for each assembled transcriptome using CEGMA[6] and BUSCO[7] with both an all-plant reference (embryophyta_odb9) and an all-eukaryote reference (eukaryota_odb9). The table below shows the percentages of complete genes found for each of the three references.at several percentiles of the whole dataset.

Percentile	CEGMA 248	BUSCO - Embryophyta*	BUSCO - Eukaryota*
5	79.03	11.2 (8.5)	66.0 (37.3)
25	89.92	44.1 (29.8)	84.9 (64.4)
50	92.34	62.5 (48.2)	90.4 (75.9)
75	93.55	75.2 (59.6)	93.7 (84.1)
95	94.76	82.6 (73.2)	96.1 (91.0)

*Complete+fragment assemblies reported with complete sequences in parentheses.

Sample purity and contamination: The diversity of sample sources ensures that there will be some contamination of the plant tissue by other environmental nucleic acids. These contaminants can reasonably be expected to include bacterial, fungal, and insect species that live in and on the plant tissues, and more rarely, from contact with larger species such as frogs, mice, birds, and humans. For most analyses these minor contaminants are not expected to have an impact, as only the most abundant of such contaminants will be present in sufficient quantities to be assembled or in many cases they were sufficiently diverged from the intended species to be easily recognized as non-plant genes. Unfortunately, this is not always the case and unfiltered contaminants were identified as long branches in the gene trees for the single copy genes (see phylogenetics section of Methods). In general, users of RNAseq data sets, including those generated for this study, should be wary of contaminant reads and assemblies derived from endogenous microbes or other sources.

We guarded against these various problems by employing several analyses, the largest of which compared the assembled sequences by Blastn to a reference set of 18S rRNA sequences from the SILVA SSU rRNA database (http://www.arb-silva.de/)[8]. For most samples we were able to establish that an 18S rDNA sequence taxonomically close to the intended species was present. In a few cases, additional 18S sequences more-closely matched to other plants were found, suggesting that these might have been contaminated by other plants. We found that 560 samples have non-plant contaminants while 108 samples had unexpected matches with other plants. Contaminant sequences were filtered and 18 samples with multiple plant contaminant sequences (18S, plastid genes and protein-coding nuclear genes) were excluded from further analyses. Possible remaining contaminant sequences identified as highly divergent either when mapping reads to plastid gene assemblies or as exceedingly long branches in gene tree phylogenies. These reads and assemblies were filtered as described below.

For the chloroplast DNA sequence analyses, contaminating chloroplast sequences proved to be common—likely due to the high copy number per cell—and appropriate steps were taken to remove contaminants which was facilitated by the large number of chloroplast reference genes and genomes already available[9]. On average, 13.4% of

sequences identified as a chloroplast gene from a particular sample were discarded as too divergent to represent the true sequence of that taxon.

Protein translation: To identify proteins within the assembled transcripts, they were passed through TransPipe[10]. The reading frame and protein translation for each sequence is identified by comparison to protein sequences from 25 sequenced and annotated plant genomes from Phytozome[11]. Using BLASTX[12], best hit proteins are paired with each gene at a minimum cutoff of 30% sequence similarity over at least 150 sites. Genes that did not have a best hit protein at this level are removed. To determine reading frame and generate estimated amino acid sequences, each gene is aligned against its best hit protein by Genewise 2.2.2[13]. Using the highest scoring Genewise DNA-protein alignments, custom Perl scripts are used to remove stop and 'N' containing codons and produce estimated amino acid sequences for each gene. Output includes paired DNA and protein sequences with the DNA sequence reading frame corresponding to each protein sequence.

Phylogenetic Analyses

Phylogenetic methods are described in the Methods section of the main text. Below are the command-line calls for the scripts and programs used for each phylogenetic analysis. All scripts used to perform analyses on the nuclear gene data are available at https://github.com/smirarab/1kp.

Sequence alignment and filtering

To build the combined PASTA and UPP alignments, we used the following script:

runupp.sh <gene_id> <num_cpus> <genes directory>

We then used scripts mask-gt.sh to mask gappy sites and sequences from alignments and used remove-long-branch.sh to mask long branches. The last script uses Dendropy15 internally.

Gene tree estimation: We used the following scripts to estimate gene trees:

- runraxml-rapidboot.sh upp-masked.fasta.mask10sites.mask33taxa FAA 4 [gene id] main 10 100 mp slow
- runraxml-rapidboot.sh upp-masked.fasta.mask10sites.mask33taxa FNA2AA 4 [gene id] main 10 100 mp slow

These scripts internally run the following RAxML commands:

- raxmlHPC-PTHREADS -m <GTRGAMMA|PROTGAMMA…> -T 4 -n best -s <alignment_file> -p <random number> -N 10
- raxmlHPC-PTHREADS -m <GTRGAMMA|PROTGAMMA…> -T 4 -n ml -s <alignment_file> -p <random number> -N 100 -b <random number>

Species tree estimation:
To estimate species trees with no contraction of polytomies, we use:
java -jar astral.5.0.3.jar -i Best -o <output>
To remove genes with fewer than half the species present, we run
java -jar astral.5.0.3.jar -i Best -o <output> -m 589
To estimate species trees with low support branches contracted, we first use:
nw_ed 'i & b<33' o Best > Best.33
to contracts branches with below 33% support from gene trees. We then use:
java -jar astral.5.0.3.jar -i Best.33 -o <output>
to infer the tree.
To score a species tree to find quartet scores per branch, we use scripts below available at https://github.com/smirarab/1kp/tree/master/scripts/hypo-test

- poly-for-hyp-4-11-02.sh: compute p-value for polytomy null hypothesis using ASTRAL (-t 10 command) for focal points of the tree
- pos-for-hyp-4-11-2.sh: compute three alternative posterior probabilities using ASTRAL (-t 4 command) for focal points of the tree
- quart-for-hyp-4-11-2.sh: compute three alternative posterior probabilities using ASTRAL (-t 8 command) for focal points of the tree

Estimation of supermatrix trees:
alltaxa
FAA-upp-masked.fasta.mask10sites.mask33taxa.bootstrap.tre
FNA2AA-upp-masked-c12.fasta.mask10sites.mask33taxa.bootstrap.tree
eudicots
eudicot.FAA-upp-masked.fasta.mask10sites.mask33taxa.bootstrap.tree
eudicot.FNA2AA-upp-masked-c12.fasta.mask10sites.mask33taxa.bootstrap.tree
remove-rogue
filtered_FAA-upp-masked.fasta.mask10sites.mask33taxa.bootstrap.tree
filtered_FNA2AA-upp-masked-c12.fasta.mask10sites.mask33taxa.bootstrap.tree

The commands used to reproduce the results are given below.

Convert the supermatrix alignment to the ExaML input format
parse-examl -s <input_alignment> -n <output_binary_file> -m <DNA|PROT> -q <partition_file>

Compute the RAxML MP starting tree
raxmlHPC-PTHREADS-SSE3 -T 12 -s <input_alignment> -p <random_seed> -m <GTRGAMMA|PROTGAMMAJTT> -n <name> –y -q <partition_file>

Compute the FastTree-2 ML starting tree
FastTreeMP (if DNA: -nt -gtr) -nosupport -out <output_tree> -nopr <input_alignment>

Compute GTR matrix and alpha parameter for codon position
raxmlHPC-PTHREADS-SSE3 -T 12 -s <input_codon_alignment> -T 6 –m GTRGAMMA -n <name> -p 1111

Generate bootstrap replicate alignments

```
raxmlHPC-PTHREADS-SSE3 -T 12 -s <input_alignment> -f j –m
<GTRGAMMA|PROTGAMMAJTT> -n <name> -q <partition_file> -b 1111 –N 100
```

Compute the ExaML final/replicate tree

```
examl-OMP -s <input_binary_file> -m GAMMA -n <name> -t <starting_tree>
```

Inferring and Placing Whole-Genome Duplications on the Phylogeny

We used a total evidence approach to infer and place ancient WGDs across the 1KP phylogeny. WGDs were inferred from age distributions of gene duplications by analyzing transcriptomes of single species with the DupPipe pipeline[10]. To place inferred WGDs from Ks plots onto the phylogeny, we compared the median Ks of these peaks to the divergence of orthologs among species across the phylogeny[10]. Finally, we employed MAPS analyses[14,15] to confirm the inference and phylogenetic placement of the ancient WGDs inferred in the preceding analyses. The detailed descriptions for each approach are provided in the methods section and results of all analyses are available in Supplementary Tables 2–5. Supplementary Table 2 provides an integrated guide to all of the results used to infer and place WGDs in the 1KP phylogeny (Fig. 4, Supplementary Fig. 8).

For each transcriptome, we used the DupPipe pipeline to construct gene families and estimate the age of gene duplications. The age distribution of gene duplications was plotted on two sets of histograms with x-axis scales of Ks = 2 and Ks = 5 to assess WGDs at different scales (Supplementary Table 2, https://bitbucket.org/barkerlab/1kp). To identify significant features in the paralog age distributions, two statistical tests were employed (K-S goodness of fit tests, and mixture models). A bootstrapped K-S goodness of fit test[16] was used to assess if the overall age distributions deviated from a simulated null without paleopolyploidizations. Taxa that significantly deviated from the null were then further explored with mixture models and manual curation to infer peaks of gene duplication consistent with WGDs. We used a mixture model implemented in the mixtools R package[17] to identify significant peaks and estimate their median Ks values (Supplementary Table 3). Although we examined Ks plots out to Ks = 5, we did not consider peaks with Ks > 2 as potential WGDs without other data available. This is because beyond Ks = 2, mixture models may identify peaks due to error rather than biological signal[18]. We also mitigated error in WGD inference from Ks plots by estimating the Ks of gene duplications from gene trees, rather pairwise comparisons[19]. Consistent with this practice, 99.6% of all inferred WGDs in our analyses had a median paralog divergence of Ks < 2 and should not be subject to significant error in their inference or be false positives from mixture model overfitting.

For many species with evidence of WGDs, Ks plots of their sister lineages did not contain evidence of the putative WGD. We inferred that these WGDs occurred independently in their lineage's history and did not occur in the ancestry of their sister lineages in the 1kp phylogeny. We further confirmed the placement of these independent WGDs with analyses of ortholog divergence (Supplementary Table 4). We expected the median Ks of the signature peak of this independent WGD should be younger than the ortholog divergence between the species with evidence of the WGD

and its sister taxa that did not. By calculating the ortholog divergence between two species from sister lineages, we estimated the expected upper bound of Ks for an independent WGD and confirmed the placement of each putative independent WGD. Comparisons of ortholog divergence and the median Ks values of peaks inferred as WGDs may be found in Supplementary Tables 2–4.

For any WGDs that may be shared by at least 3 species, we further used MAPS[14,15] to confirm the placement of the shared WGD(s) (Supplementary Tables 2, 5-6). We circumscribed and constructed nuclear gene family phylogenies from multiple species for each MAPS analysis. MAPS requires nuclear gene families to contain at least one gene copy from each taxon in a given MAPS analysis and discards the remaining gene family clusters. To maintain sufficient gene tree numbers for each MAPS analysis, we used collections of gene family phylogenies for six to eight taxa to infer ancient WGDs. MAPS identifies and records nodes with a gene duplication shared by descendant taxa.

Errors in transcriptome or genome assembly, gene family clustering, and the construction of gene family phylogenies can result in topological errors in gene trees[20]. Previous studies have suggested that errors in gene trees can lead to bias placements of duplicates towards the root of the tree and losses towards the tips of the tree[21]. For this reason, we aim to put focal nodes for a particular MAPS analysis test in the middle of the phylogeny. To further decrease potential error in our inferences of gene duplications, we required at least 45% of the ingroup taxa to be present in all subtrees analyzed by MAPS. If this minimum ingroup taxa number requirement is not met, the gene subtree will be filtered out and excluded from our analysis. Increasing taxon occupancy should lead to more accurate duplication inference and reduce some of the biases in mapping duplications onto a species tree[21,22].

We expect that the number of gene duplications on each branch will vary with branch length. We also expect that changes in species composition among our MAPS analyses will impact the number of shared gene duplications observed at a particular branch in different analyses. To account for this variation in our comparisons, all MAPS analyses were first compared to a null simulation of the number of gene duplications we expect on each branch from background gene birth and death. A Fisher's exact test, implemented in R was used to identify locations with significant increases of gene duplication compared with a null simulation (Supplementary Table 5). For all MAPS analyses with significant bursts of gene duplication detected with the Fisher's exact test, we simulated an additional distribution of gene trees with a WGD at the location of the significant burst of duplication. If these increases in gene duplications are due to WGDs, we expect the number of shared gene duplication in the observed MAPS result to be consistent with these positive simulations. A second Fisher's exact test was then used to characterize bursts of duplication as WGDs if the observed data was not significantly less than the percentage of duplications in a positive simulation (Supplementary Table 6).

For the null simulations, we first estimated the mean background gene duplication rate (λ) and gene loss rate (μ) with WGDgc[23]. Gene count data were obtained from

OrthoFinder[24] clusters associated with each species tree (SupplementaryTable 11). λ and μ were estimated using only gene clusters that spanned the root of their respective species trees, which has been shown to reduce biases in the maximum likelihood estimates of λ and μ[23]. We chose a maximum gene family size of 100 for parameter estimation, which was necessary to provide an upper bound for numerical integration of node states[23]. We provided a prior probability distribution on the number of genes at the root of each species tree, such that ancestral gene family sizes followed a shifted geometric distribution with mean equal to the average number of genes per gene family across species (Supplementary Table 11).

Gene trees were then simulated within each MAPS species trees using the GuestTreeGen program from GenPhyloData[25]. For each species tree, we simulated 3000 gene trees with at least one tip per species: 1000 gene trees at the λ and μ maximum likelihood estimates, 1000 gene trees at half the estimated λ and μ, 1000 trees at three times λ and μ. For all simulations, we applied the same empirical prior used for estimation of λ and μ. We then randomly resampled 1000 trees without replacement from the total pool of gene trees 100 times to provide a measure of uncertainty on the percentage of subtrees at each node. Given that natural gene tree discordance and errors from methodological sources are an inescapable part of our analyses, we also introduced error into our gene tree simulations. For each set of 1000 gene trees, each gene tree had a 50% of chance that three pairs of genes would randomly swap positions. We selected to randomize three pairs of genes to introduce a range of errors in our gene trees similar to other studies[26].

For positive simulations, we simulated gene trees using the same methods described above. However, we incorporated a WGD at the location in each of the MAPS phylogenies with significantly larger numbers of gene duplications compared to the null simulation. Previous empirical estimates of paralogs retained following a plant WGD are approximately 10% on average[27]. To be conservative for inferring WGDs in our MAPS analyses, we allowed at least 20% of the genes to be retained following the simulated WGD to account for biased gene retention and loss. To assess power for WGDs with lower gene retention rates, we ran an additional simulation using 15% gene retention (Supplementary Table 6).

To place ancestrally shared WGDs on species phylogenies, we used simulated gene family phylogenies with and without WGDs to identify significant bursts of gene duplication that are consistent with WGDs. A WGD will produce a large burst of shared duplications across taxa and gene trees. This burst of duplication will appear as an increase in the percentage of shared gene duplications at a particular branch in our MAPS analyses. We interpreted an episodic burst of shared gene duplication that had significantly more shared duplications than expected compared to the null simulations and statistically consistent with our positive simulations of WGDs as evidence of a shared WGD. Combined with evidence from Ks plots and ortholog divergence analyses, the MAPS analyses provided phylogenomic tests for evidence of ancient, shared genome duplication events (Supplementary Table 2). In the few cases where our data conflicted, we used a total evidence approach to infer duplication events. These were

mostly cases where inferences from Ks plots, ortholog comparisons, and the previous literature agreed, but MAPS did not. In these instances where MAPS inferred a significant burst of gene duplication not clearly apparent in other analyses, we indicated this in Supplementary Tables 6 and as blue squares on the ED WGD Phylogeny Figure.

All datasets used for the WGD analyses are available at https://bitbucket.org/barkerlab/1kp and scripts used for the MAPS analyses are at https://bitbucket.org/barkerlab/maps .

Gene Family Evolution

Transcriptome completeness for gene family analyses

We used the BUSCO[7] assessments described above for the eukaryote data set (eukaryota_odb9) with conditional reciprocal best BLAST (CRBB) hits calculated using CRB-BLAST [28] with default parameters for 248 core eukaryotic genes (CEGs) distributed with the CEGMA software (Core Eukaryotic Genes Mapping Approach)[29]. CRBB hits were produced for each library individually which accounts for species specific threshold determination.

Percentage BUSCO abundance was calculated as 100% minus missing percentage. Percentage CEG abundance was calculated as number of CEGs with a CRBB hit divided by the total number of used CEGs. All libraries that showed less than 57.5% BUSCO abundance were removed from further analysis to account for possible transcriptome incompleteness due to library preparation or sample quality.

Pfam detection is not unduly biased by the prevalence of angiosperm exemplars

PFAM HMMs[30] constructed from manually validated seed sequences in the UniProt database. Unfortunately, these seed sequences are highly biased towards angiosperms, since *A. thaliana* is a popular model in plant genetics. For example, the validated sequence collection for the F-box, a domain found across plants, animals, and fungi, has 158 sequences from Viridiplantae of which all come from angiosperms and 155 alone from *A. thaliana*.

To check whether the sequence composition biases the results of this study towards angiosperms and is the reason for the trends observed in Fig 4b we compared the results of: a) the PFAM database, b) the PFAM database without sequences from streptophytes. Default settings of HMMER v3.1b2[31] were used to screen for domain hits. Some gene families have been identified with using custom HMMs and therefore were not included in this study. Further, HMMs with less than 6 sequences left after eliminating streptophyte sequences have also been removed from this study. For the following 15 remaining gene families we re-evaluated family sizes: “F-box”, “AP2_EREBP”, “bHLH”, “bZIP”, “C2H2”, “C3H”, “WRKY”, “ABC”, “PP2C”, “Receptor-like Kinases”, “P450s”, “MYB”, “NAC”, “PHD”, and “GRAS”. Results from both approaches show a high Pearson correlation (r=0.997). We therefore conclude that angiosperm bias for PFAM sequence alignments cannot be responsible for the effects observed in Fig. 5b.

Evolution of MADS-box gene evolution

Identification of MADS-box genes: 1KP transcripts were translated in all six possible reading frames and searched using the HMMer program[31] and the HMM of the MADS domain as described by Gramzow and Theißen[32]. As some of the transcripts are incomplete and as the K domain is a second characteristic protein domain of MIKC-type MADS domain proteins[33], we also conducted HMMer searches using a customized HMM of the K domain. An alignment of exemplary K domains from MIKC-type MADS-domain proteins was used to identify transcript assemblies including only the K-domain (Extended Data Fig. 1). The alignment was used to construct an HMM for the K domain.

Phylogeny of MADS-box genes: $MIKC^C$-group MADS-domain proteins from non-algae and non-seed-plant species as found in the 1kp data, known MADS-domain proteins from land plants, and CgMADS1 from *Chara globularis* were aligned using Probalign[34]. The alignment was checked manually, and poorly conserved positions were deleted. The MADS-box gene phylogeny (Extended Data Fig. 2) was constructed using RAxML where the protein substitution model LG[35] was chosen, the maximum likelihood phylogeny and 1000 bootstrap replicates were calculated in one program run (option "-f a"), and CgMADS1 was set as representative of the outgroup.

Supplemental Results

Species Tree Estimations

As described above, species trees were estimated using the ASTRAL gene tree summary method (Supplementary Fig. 1), and RAxML analyses of concatenated supermatrices (Supplementary Fig. 2) for the 410 single-copy nuclear genes for 1147 accessions included in Supplementary Table 1. A tree was also estimated from plastid gene sequences extracted from the RNA seq data (see methods above; Supplementary Fig. 3). Occupancy in the data matrices was high with a across species median of 339 of the 410 genes included in the ASTRAL and supermatrix analyses (Supplementary Figures 4-5). Estimated species relationships were generally robust and consistent among analyses, but resolution of some historically problematic relationships (e.g. bryophytes (Supplementary Fig. 6b) or conifer lineages and gnetales (Supplementary Fig. 6c) did vary among between ASTAL and supermatrix analyses or between amino acid and nucleotide analyses (Supplementary Fig. 6). Differences between amino acid and nucleotide analyses may be due to variation in codon usage and GC content at 1st and 3rd codon positions (Supplementary Fig. 7)

Inferred WGDs

Overall, we inferred a total of 244 potential WGD events throughout the green plant phylogeny (Fig. 4, Supplementary Fig. 8). These putative WGDs were inferred using a total evidence approach that combined inferences of WGDs from gene age distributions (Ks plots) and their phylogenetic placement based on pairwise comparisons of ortholog divergence [10,16,19]. These results were compared with simultaneous inference and phylogenetic placement of putative WGDs in MAPS[15,36]. We also compared our results to the large body of literature on WGDs during plant evolution[16,19,37–84]. We found that 106 of the 244 WGDs we inferred were previously reported in the literature (Supplementary Table 2). Almost all of the 138 newly inferred WGDs were inferred in lineages newly sampled by the 1KP project. All of the inferred WGDs were detected in Ks plots of species (Supplementary Table 3), and 63 WGDs on internal branches were also supported by MAPS analyses (Supplementary Table 2, 5-6). Nearly all of the putative WGDs were inferred with paralog peak medians of Ks < 2. These inferences should not be subject to significant error in their inference from saturation effects[16,18,81]. We found that 237 of the 244 inferred WGDs had a median peak paralog divergence of Ks < 2 in all species surveyed. Only seven of the inferred WGDs, or 2.9% of our results, had a median paralog divergence of Ks > 2 in some species. Of these seven WGDs, only 1 of the WGD inferences was higher than Ks = 2 in all species analyzed. In this case, CHROα, the WGD had an inferred median Ks ~ 2.1 in the Chloromonas algae and support from MAPS. In the other seven cases, the WGD had a Ks peak < 2 in one or more species. Two of these WGDs are also well described from previous studies using synteny (the eudicot hexaploidy ARTHγ[46,52,85,86] and the τ WGD of the monocots ORSAγ[64]), one is described with support from synteny from the first sequenced fern genomes (CYATγ in Li et al. 2018 Nature Plants), and one is well known from previous analyses and well debated (the seed plant WGD GINKα[15,44,45,87]). Only three of these very ancient WGD inferences were not previously observed. All three of these WGDs—CHROα, OSMNβ, and LYCOβ— are from newly sequenced lineages in the 1KP project. We believe these three inferences of deep WGDs from combined MAPS, Ks plots, and orthologous divergence are robust, but as with any inference future analyses with new data and/or approaches may revise the nature of the duplications or their phylogenetic placements.

Our analyses with MAPS also found evidence of 24 significant bursts of gene duplication that were not clearly associated with a WGD from the literature or our analyses of Ks plots. These are indicated in the Supplementary Fig. 8 as blue squares and noted in the associated MAPS analyses as significant duplications without a WGD designation (Supplementary Table 6). Among all of these bursts of duplication, most occur among the mosses, monocots, and the Lamiales (Supplementary Fig. 8). It may be that our species tree has more error in these regions of the phylogeny and or that gene birth/death rates are highly heterogeneous among these taxa. Both issues could interfere with our MAPS inferences. Further analyses are needed to better resolve the nature of these significant duplications in MAPS and the placements of inferred WGDs in these regions of the phylogeny.

Supplementary Fig. 1 Phylogenetic inferences based on ASTRAL analysis of 410 single-copy nuclear gene families extracted from genome and transcriptome data from 1178 species, including 1112 green plant (Viridiplantae) species. Branches with BS support below 33% were removed from the gene trees before estimating the ASTRAL tree. Branch support measured using local posterior probability (localPP) is shown on branches with support less than 1.0. Nodes with support less than 1.0 are marked with a circle. Supplementary Table 1 includes taxonomy for each species and mapping of binomial species names to four letter codes. This information and major group names (colors; see columns "Code" and "Brief Classification" in misc/names.csv), and data used to build the figure, available at doi.org/10.5281/zenodo.3255100

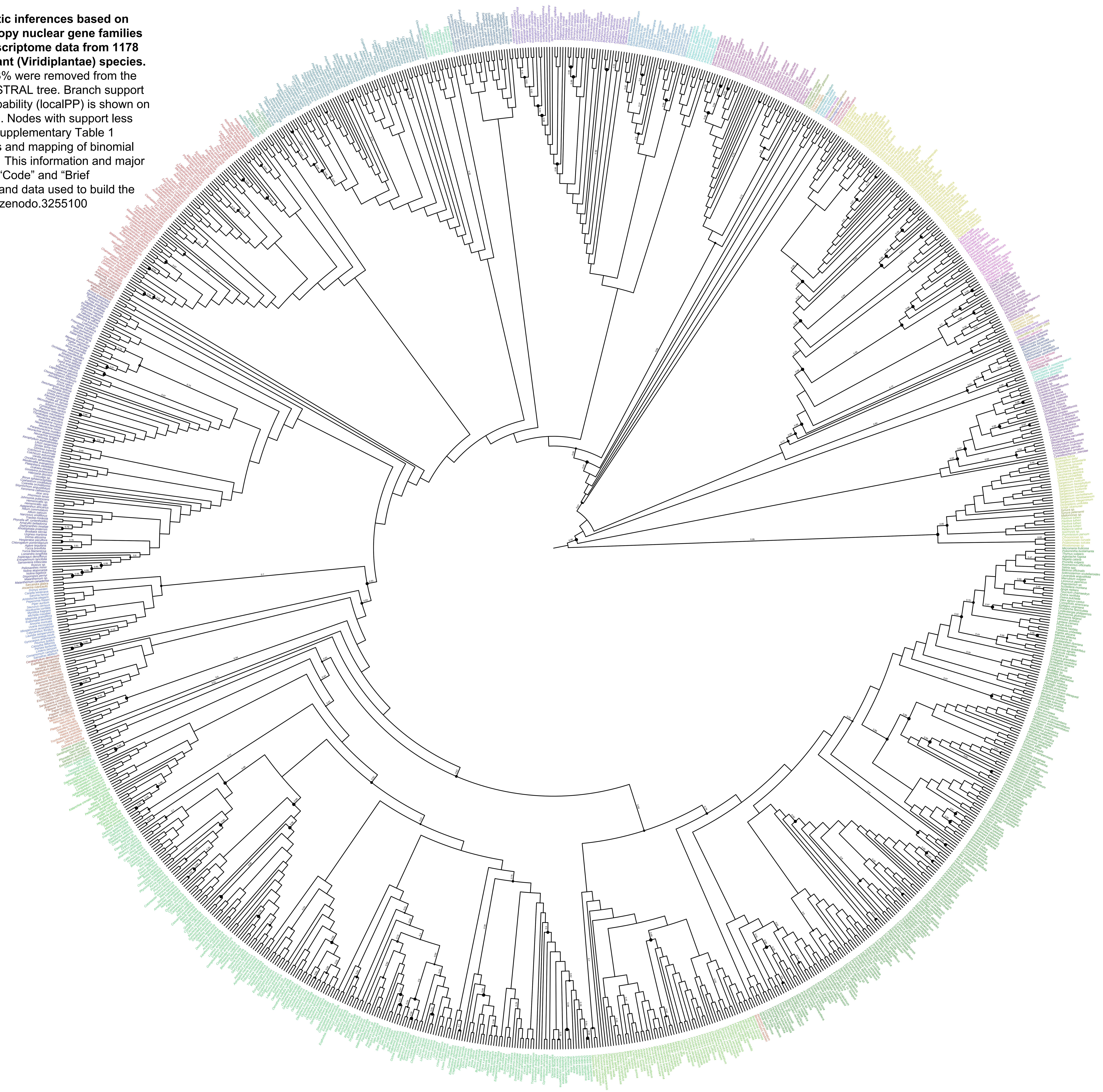

Supplementary Fig. 2 Phylogenetic inferences in a concatenated analysis of 410 single-copy nuclear gene families extracted from genome and transcriptome data from 1178 species, including 1112 green plant (Viridiplantae) species. Trees were inferred using ExaML. Branch support measured using bootstrap support (100 replicates) is shown on branches with support less than 100%. Supplementary Table 1 includes taxonomy for each species and mapping of binomial species names to four letter codes. This information and major group names (colors; see columns "Code" and "Brief Classification" in misc/names.csv), and data used to build the figure, available at doi.org/10.5281/zenodo.3255100.

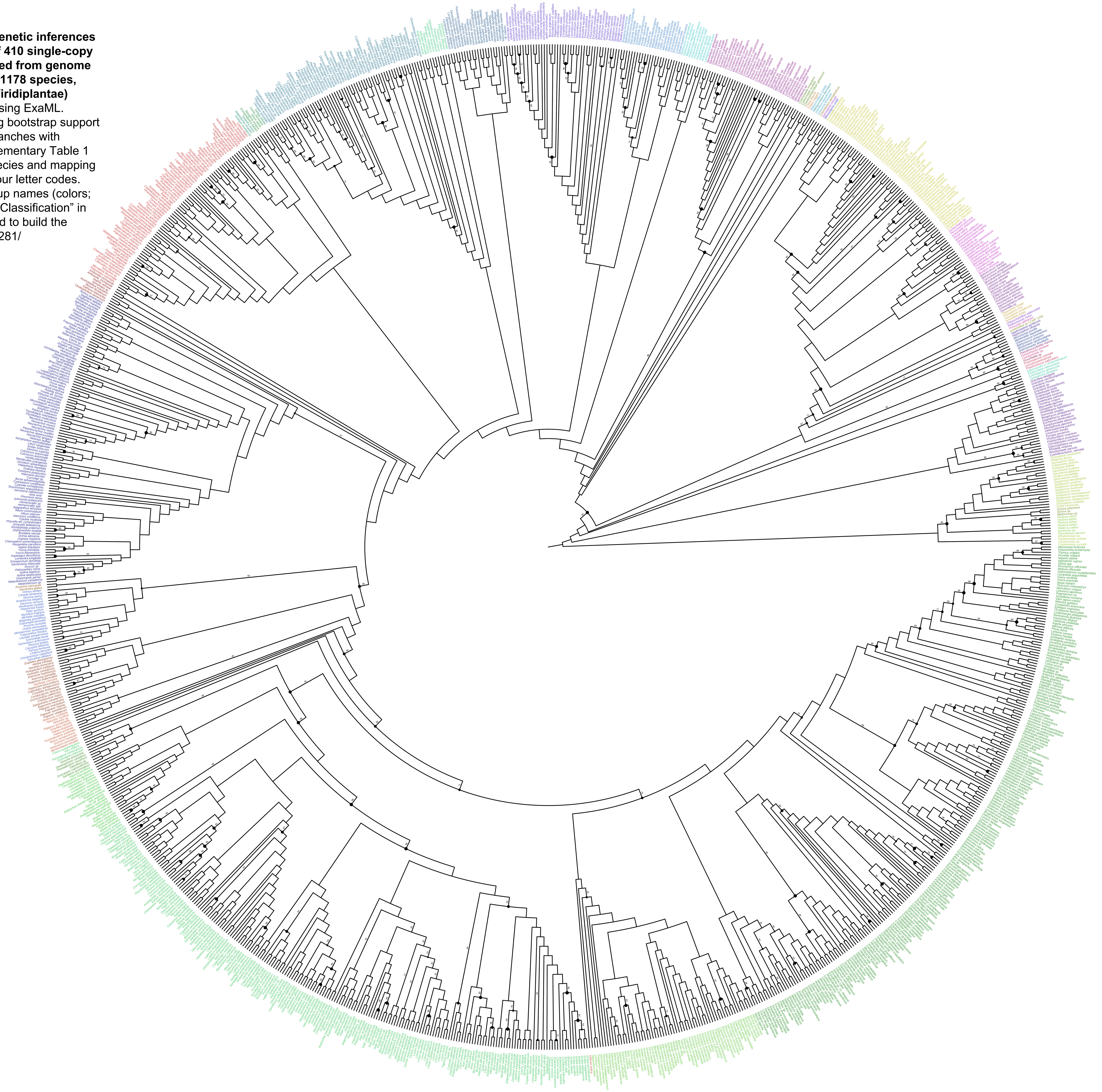

Supplementary Fig. 3 Phylogenetic inferences derived from ExaML analyses of a concatenated supermatrix of amino acid alignments for 78 shared protein-coding genes as described by Gitzendanner et al.[72]. Bootstrap support (100 replicates) is shown on branches with support less than 100% and associated have widths proportional to the support values (if < 100%). Major groups are differentiated by tip colors, identically to Supplementary Figs 1& 2. Supplementary Table 1 includes taxonomy for each species and mapping of bionomial species names to four letter codes. This information and major group names (colors; see columns “Code” and “Brief Classification” in misc/names.csv), and data used to build the figure, available at doi.org/10.5281/zenodo.3255100..

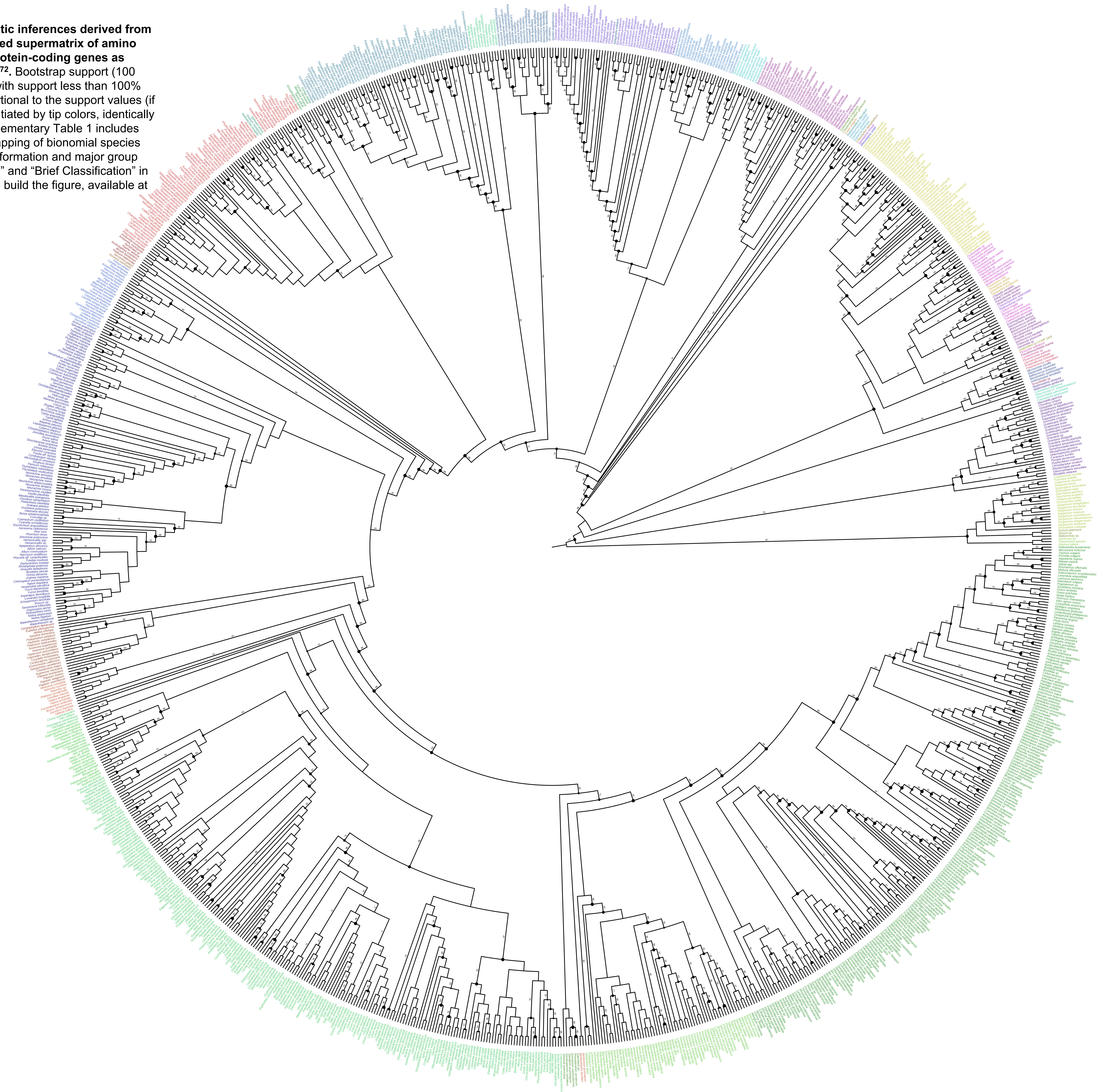

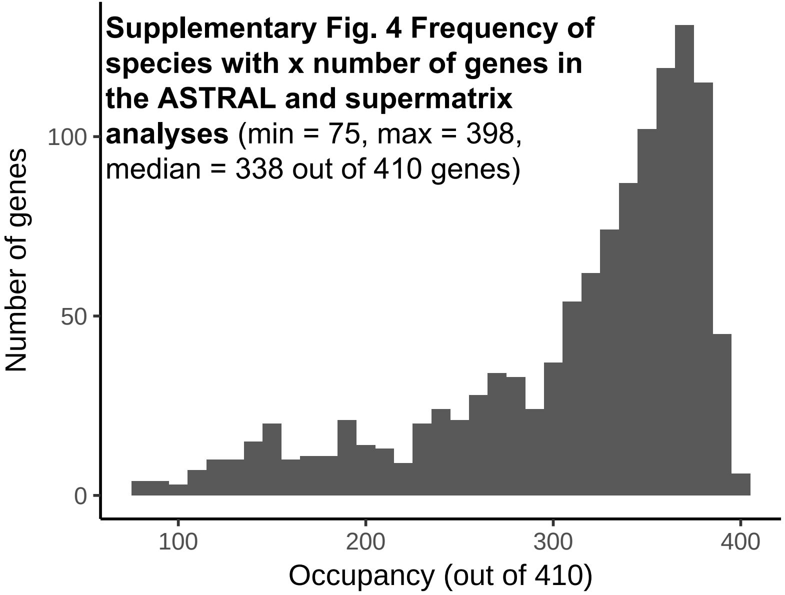

Supplementary Fig. 4 Frequency of species with x number of genes in the ASTRAL and supermatrix analyses (min = 75, max = 398, median = 338 out of 410 genes)

Supplementary Fig. 5 Species (X-axis) occupancy in single copy gene alignments (Y-axis). Species names corresponding to the x-axis numbered taxon codes and data are included in Supplementary Table 1 and posted at doi.org/10.5281/zenodo.3255100

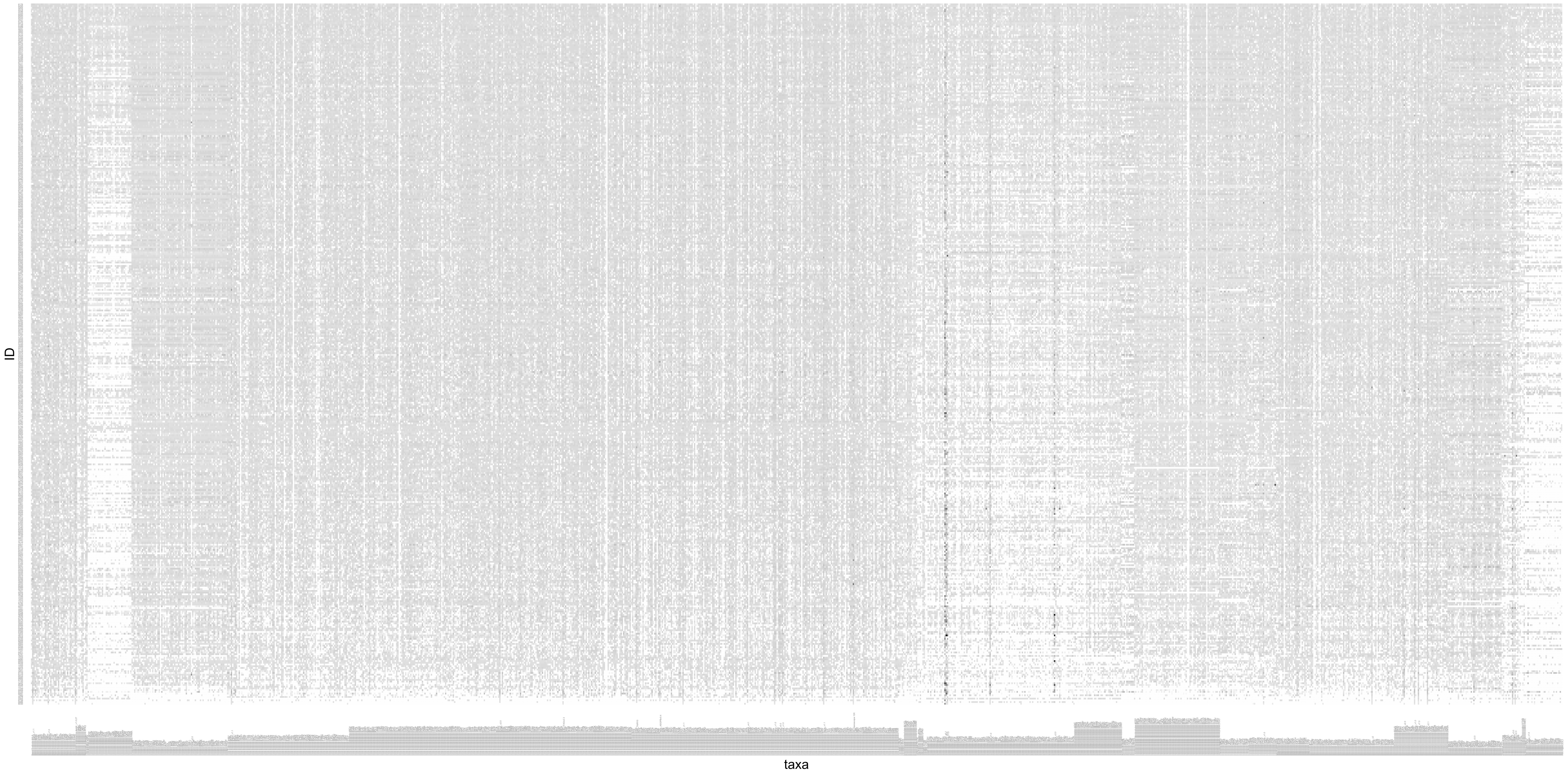

Supplementary Fig. 6 Support for alternative resolutions for problematic portions of the green plant phylogeny. Rows represent hypothesized branching orders, and columns correspond to various analyses, including ASTRAL trees with various levels of contraction within gene trees, ("full for no contraction or polytomies contracting clades with less than 33%, 20%, 10%, 5% bootstrap support), ASTRAL on binned gene trees, and concatenation using ExaML (CAML), applied to both amino acid data (AA) and DNA sequences, with third codon position removed (C12). Also shown are trees with rogue taxa (*Dillenia indica, Tetrastigma obtectum, Tetrastigma voinierianum, Vitis vinifera, Cissus quadrangularis, Ceratophyllum demersum, "Spirotaenia" sp.*, and *Prasinococcus capsulatus*) removed and with only eudicots included. Shades of blue correspond to clades that have been recovered in an analysis, and the light to dark shade indicates strength of support (local posterior probability for ASTRAL and bootstrap for CAML). Yellow indicates a clade was not recovered, but light (dark) yellow corresponds to branches that were (were not) compatible with that clades if branches below 90% support were contracted. Missing tiles (e.g., those in white) represent hypotheses that could not be tested because relevant taxa were missing. The figures are created using DiscoVista[88]. For data used to build the figure, refer to doi.org/10.5281/zenodo.3255100

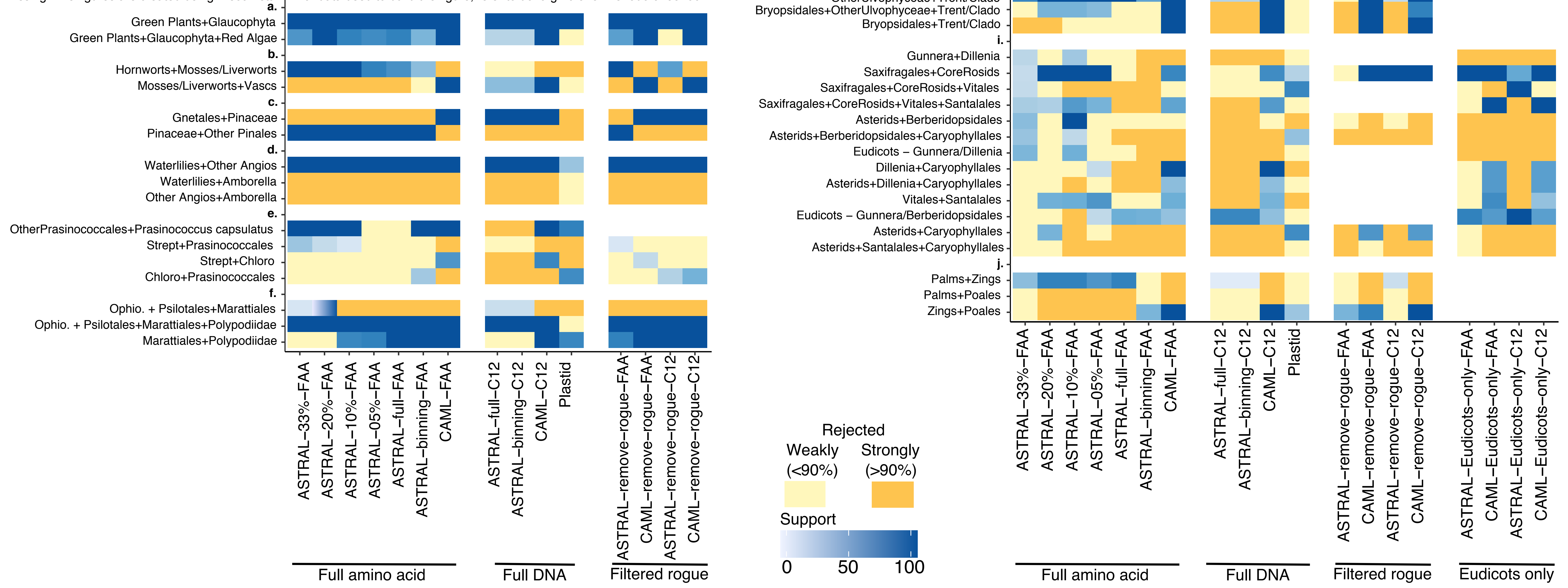

Supplementary Fig. 7 Distribution of the GC content across all single-copy genes for each species (x-axis). The distributions are shown for all codon positions (top row), and each also separately for each of the codon positions (rows 2-4). The GC content of the first and the second codon position are on average similar across all species, whereas, the GC content of the third codon varies dramatically, rendering assumptions of the GTR model invalid. The x-axis is labeled by numbers with four letter taxon codes and species names posted at doi.org/10.5281/zenodo.3255100

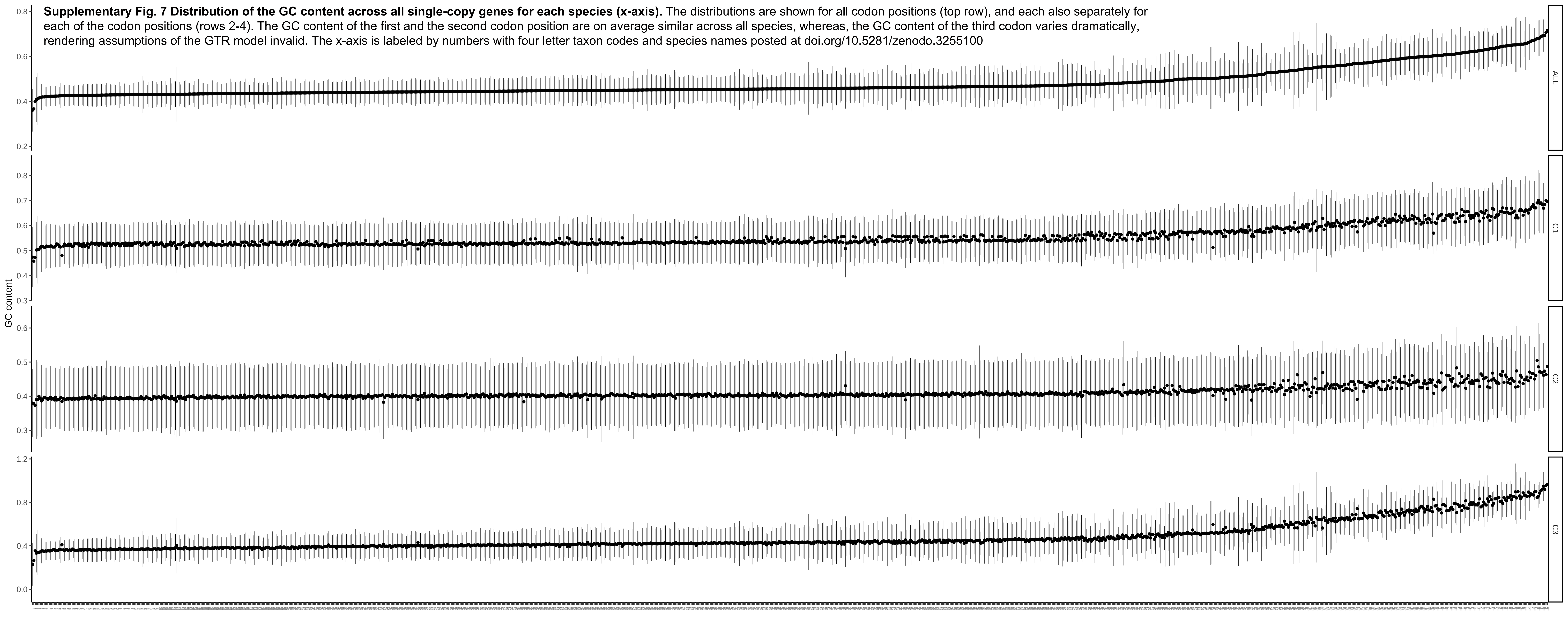

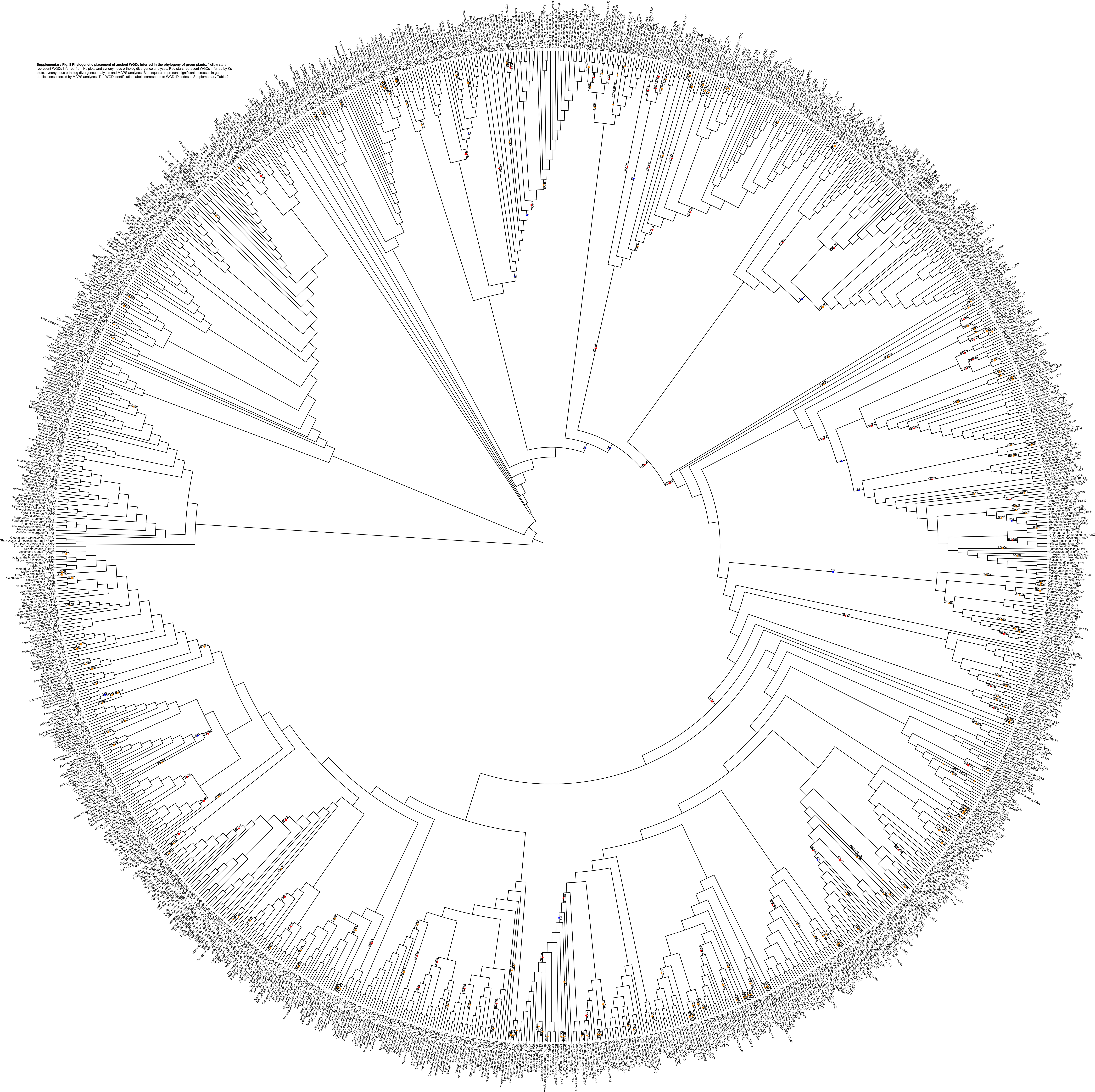

Supplementary Fig. 8 Phylogenetic placement of ancient WGDs inferred in the phylogeny of green plants. Yellow stars represent WGDs inferred from Ks plots and synonymous ortholog divergence analyses; Red stars represent WGDs inferred by Ks plots, synonymous ortholog divergence analyses and MAPS analyses; Blue squares represent significant increases in gene duplications inferred by MAPS analyses; The WGD identification labels correspond to WGD ID codes in Supplementary Table 2.

Supplemental References:

1. Boyle, B. *et al.* The taxonomic name resolution service: an online tool for automated standardization of plant names. *BMC Bioinformatics* **14**, 16 (2013).
2. Johnson, M. T. J. *et al.* Evaluating methods for isolating total RNA and predicting the success of sequencing phylogenetically diverse plant transcriptomes. *PLoS One* **7**, e50226 (2012).
3. Jordon-Thaden, I. E., Chanderbali, A. S., Gitzendanner, M. A. & Soltis, D. E. Modified CTAB and TRIzol protocols improve RNA extraction from chemically complex Embryophyta. *Appl. Plant Sci.* **3**, (2015).
4. Xie, Y. *et al.* SOAPdenovo-Trans: de novo transcriptome assembly with short RNA-Seq reads. *Bioinformatics* **30**, 1660–1666 (2014).
5. Smith-Unna, R., Boursnell, C., Patro, R., Hibberd, J. M. & Kelly, S. TransRate: reference-free quality assessment of de novo transcriptome assemblies. *Genome Res.* **26**, 1134–1144 (2016).
6. Parra, G., Bradnam, K. & Korf, I. CEGMA: a pipeline to accurately annotate core genes in eukaryotic genomes. *Bioinformatics* **23**, 1061–1067 (2007).
7. Simão, F. A., Waterhouse, R. M., Ioannidis, P., Kriventseva, E. V. & Zdobnov, E. M. BUSCO: assessing genome assembly and annotation completeness with single-copy orthologs. *Bioinformatics* **31**, 3210–3212 (2015).
8. Quast, C. *et al.* The SILVA ribosomal RNA gene database project: improved data processing and web-based tools. *Nucleic Acids Res.* **41**, D590–6 (2013).
9. Gitzendanner, M. A., Soltis, P. S., Wong, G. K.-S., Ruhfel, B. R. & Soltis, D. E. Plastid phylogenomic analysis of green plants: A billion years of evolutionary history. *Am. J. Bot.* **105**, 291–301 (2018).
10. Barker, M. S. *et al.* EvoPipes.net: Bioinformatic Tools for Ecological and Evolutionary Genomics. *Evol. Bioinform. Online* **6**, 143–149 (2010).

11. Goodstein, D. M. *et al.* Phytozome: a comparative platform for green plant genomics. *Nucleic Acids Res.* **40**, D1178–86 (2012).

12. Wheeler, D. L. *et al.* Database resources of the National Center for Biotechnology Information. *Nucleic Acids Res.* **36**, D13–21 (2008).

13. Birney, E., Clamp, M. & Durbin, R. GeneWise and Genomewise. *Genome Res.* **14**, 988–995 (2004).

14. Li, Z. *et al.* Multiple large-scale gene and genome duplications during the evolution of hexapods. *Proc. Natl. Acad. Sci. U. S. A.* **115**, 4713–4718 (2018).

15. Li, Z. *et al.* Early genome duplications in conifers and other seed plants. *Sci Adv* **1**, e1501084 (2015).

16. Cui, L. *et al.* Widespread genome duplications throughout the history of flowering plants. *Genome Res.* **16**, 738–749 (2006).

17. Benaglia, T., Chauveau, D., Hunter, D. R. & Young, D. mixtools: AnRPackage for Analyzing Finite Mixture Models. *Journal of Statistical Software* **32**, (2009).

18. Vanneste, K., Van de Peer, Y. & Maere, S. Inference of genome duplications from age distributions revisited. *Mol. Biol. Evol.* **30**, 177–190 (2013).

19. Barker, M. S. *et al.* Multiple paleopolyploidizations during the evolution of the Compositae reveal parallel patterns of duplicate gene retention after millions of years. *Mol. Biol. Evol.* **25**, 2445–2455 (2008).

20. Yang, Y. & Smith, S. A. Orthology inference in nonmodel organisms using transcriptomes and low-coverage genomes: improving accuracy and matrix occupancy for phylogenomics. *Mol. Biol. Evol.* **31**, 3081–3092 (2014).

21. Hahn, M. W. Bias in phylogenetic tree reconciliation methods: implications for vertebrate genome evolution. *Genome Biol.* **8**, R141 (2007).

22. Smith, S. A., Moore, M. J., Brown, J. W. & Yang, Y. Analysis of phylogenomic datasets reveals conflict, concordance, and gene duplications with examples from animals and

plants. *BMC Evol. Biol.* **15**, 150 (2015).

23. Rabier, C.-E., Ta, T. & Ané, C. Detecting and locating whole genome duplications on a phylogeny: a probabilistic approach. *Mol. Biol. Evol.* **31**, 750–762 (2014).
24. Emms, D. M. & Kelly, S. OrthoFinder: solving fundamental biases in whole genome comparisons dramatically improves orthogroup inference accuracy. *Genome Biol.* **16**, 157 (2015).
25. Sjöstrand, J., Arvestad, L., Lagergren, J. & Sennblad, B. GenPhyloData: realistic simulation of gene family evolution. *BMC Bioinformatics* **14**, 209 (2013).
26. Wu, D., Jospin, G. & Eisen, J. A. Systematic Identification of Gene Families for Use as 'Markers' for Phylogenetic and Phylogeny-Driven Ecological Studies of Bacteria and Archaea and Their Major Subgroups. *PLoS One* **8**, e77033 (2013).
27. Tiley, G. P., Ané, C. & Burleigh, J. G. Evaluating and Characterizing Ancient Whole-Genome Duplications in Plants with Gene Count Data. *Genome Biol. Evol.* **8**, 1023–1037 (2016).
28. Aubry, S., Kelly, S., Kümpers, B. M. C., Smith-Unna, R. D. & Hibberd, J. M. Correction: Deep Evolutionary Comparison of Gene Expression Identifies Parallel Recruitment of Trans-Factors in Two Independent Origins of C4 Photosynthesis. *PLoS Genet.* **12**, e1006087 (2016).
29. Parra, G., Bradnam, K., Ning, Z., Keane, T. & Korf, I. Assessing the gene space in draft genomes. *Nucleic Acids Res.* **37**, 289–297 (2009).
30. Finn, R. D. *et al.* The Pfam protein families database: towards a more sustainable future. *Nucleic Acids Res.* **44**, D279–85 (2016).
31. Eddy, S. R. Accelerated Profile HMM Searches. *PLoS Comput. Biol.* **7**, e1002195 (2011).
32. Gramzow, L. & Theißen, G. Phylogenomics of MADS-Box Genes in Plants - Two Opposing Life Styles in One Gene Family. *Biology* **2**, 1150–1164 (2013).
33. Ma, H., Yanofsky, M. F. & Meyerowitz, E. M. AGL1-AGL6, an Arabidopsis gene family with

similarity to floral homeotic and transcription factor genes. *Genes Dev.* **5**, 484–495 (1991).

34. Roshan, U. & Livesay, D. R. Probalign: multiple sequence alignment using partition function posterior probabilities. *Bioinformatics* **22**, 2715–2721 (2006).
35. Le, S. Q. & Gascuel, O. An improved general amino acid replacement matrix. *Mol. Biol. Evol.* **25**, 1307–1320 (2008).
36. Li, Z. *et al.* Multiple large-scale gene and genome duplications during the evolution of hexapods. *Proc. Natl. Acad. Sci. U. S. A.* **115**, 4713–4718 (2018).
37. Landis, J. B. *et al.* Impact of whole-genome duplication events on diversification rates in angiosperms. *Am. J. Bot.* **105**, 348–363 (2018).
38. Shi, T., Huang, H. & Barker, M. S. Ancient genome duplications during the evolution of kiwifruit (Actinidia) and related Ericales. *Ann. Bot.* **106**, 497–504 (2010).
39. Banks, J. A. *et al.* The Selaginella genome identifies genetic changes associated with the evolution of vascular plants. *Science* **332**, 960–963 (2011).
40. Yang, Y. *et al.* Dissecting Molecular Evolution in the Highly Diverse Plant Clade Caryophyllales Using Transcriptome Sequencing. *Mol. Biol. Evol.* **32**, 2001–2014 (2015).
41. Ibarra-Laclette, E. *et al.* Architecture and evolution of a minute plant genome. *Nature* **498**, 94–98 (2013).
42. Barker, M. S., Vogel, H. & Schranz, M. E. Paleopolyploidy in the Brassicales: analyses of the Cleome transcriptome elucidate the history of genome duplications in Arabidopsis and other Brassicales. *Genome Biol. Evol.* **1**, 391–399 (2009).
43. Edger, P. P. *et al.* The butterfly plant arms-race escalated by gene and genome duplications. *Proc. Natl. Acad. Sci. U. S. A.* **112**, 8362–8366 (2015).
44. Ruprecht, C. *et al.* Revisiting ancestral polyploidy in plants. *Sci Adv* **3**, e1603195 (2017).
45. Jiao, Y. *et al.* Ancestral polyploidy in seed plants and angiosperms. *Nature* **473**, 97–100 (2011).
46. Jiao, Y. *et al.* A genome triplication associated with early diversification of the core

eudicots. *Genome Biol.* **13**, R3 (2012).

47. Rensing, S. A. *et al.* An ancient genome duplication contributed to the abundance of metabolic genes in the moss Physcomitrella patens. *BMC Evol. Biol.* **7**, 130 (2007).
48. Rensing, S. A. *et al.* The Physcomitrella genome reveals evolutionary insights into the conquest of land by plants. *Science* **319**, 64–69 (2008).
49. Myburg, A. A. *et al.* The genome of Eucalyptus grandis. *Nature* **510**, 356–362 (2014).
50. Velasco, R. *et al.* The genome of the domesticated apple (Malus × domestica Borkh.). *Nat. Genet.* **42**, 833–839 (2010).
51. Tuskan, G. A. *et al.* The genome of black cottonwood, Populus trichocarpa (Torr. & Gray). *Science* **313**, 1596–1604 (2006).
52. Vekemans, D. *et al.* Gamma paleohexaploidy in the stem lineage of core eudicots: significance for MADS-box gene and species diversification. *Mol. Biol. Evol.* **29**, 3793–3806 (2012).
53. Ren, R. *et al.* Widespread Whole Genome Duplications Contribute to Genome Complexity and Species Diversity in Angiosperms. *Mol. Plant* **11**, 414–428 (2018).
54. Cannon, S. B. *et al.* Multiple polyploidy events in the early radiation of nodulating and nonnodulating legumes. *Mol. Biol. Evol.* **32**, 193–210 (2015).
55. Barker, M. S., Husband, B. C. & Pires, J. C. Spreading Winge and flying high: The evolutionary importance of polyploidy after a century of study. *Am. J. Bot.* **103**, 1139–1145 (2016).
56. Barker, M. S. *et al.* Most Compositae (Asteraceae) are descendants of a paleohexaploid and all share a paleotetraploid ancestor with the Calyceraceae. *Am. J. Bot.* **103**, 1203–1211 (2016).
57. Wan, T. *et al.* A genome for gnetophytes and early evolution of seed plants. *Nat Plants* **4**, 82–89 (2018).
58. Xiang, Y. *et al.* Evolution of Rosaceae Fruit Types Based on Nuclear Phylogeny in the

Context of Geological Times and Genome Duplication. *Mol. Biol. Evol.* **34**, 262–281 (2017).

59. Huang, C.-H. *et al.* Multiple Polyploidization Events across Asteraceae with Two Nested Events in the Early History Revealed by Nuclear Phylogenomics. *Mol. Biol. Evol.* **33**, 2820–2835 (2016).
60. Mandáková, T., Li, Z., Barker, M. S. & Lysak, M. A. Diverse genome organization following 13 independent mesopolyploid events in Brassicaceae contrasts with convergent patterns of gene retention. *Plant J.* **91**, 3–21 (2017).
61. Kagale, S. *et al.* Polyploid evolution of the Brassicaceae during the Cenozoic era. *Plant Cell* **26**, 2777–2791 (2014).
62. Unruh, S. A. *et al.* Phylotranscriptomic analysis and genome evolution of the Cypripedioideae (Orchidaceae). *Am. J. Bot.* **105**, 631–640 (2018).
63. McKain, M. R. *et al.* A Phylogenomic Assessment of Ancient Polyploidy and Genome Evolution across the Poales. *Genome Biol. Evol.* **8**, 1150–1164 (2016).
64. Jiao, Y., Li, J., Tang, H. & Paterson, A. H. Integrated syntenic and phylogenomic analyses reveal an ancient genome duplication in monocots. *Plant Cell* **26**, 2792–2802 (2014).
65. D'Hont, A. *et al.* The banana (Musa acuminata) genome and the evolution of monocotyledonous plants. *Nature* **488**, 213–217 (2012).
66. McKain, M. R. *et al.* Phylogenomic analysis of transcriptome data elucidates co-occurrence of a paleopolyploid event and the origin of bimodal karyotypes in Agavoideae (Asparagaceae). *Am. J. Bot.* **99**, 397–406 (2012).
67. Vanneste, K., Sterck, L., Myburg, A. A., Van de Peer, Y. & Mizrachi, E. Horsetails Are Ancient Polyploids: Evidence from Equisetum giganteum. *Plant Cell* **27**, 1567–1578 (2015).
68. Iorizzo, M. *et al.* A high-quality carrot genome assembly provides new insights into carotenoid accumulation and asterid genome evolution. *Nat. Genet.* **48**, 657–666 (2016).
69. Lee, H. *et al.* The Genome of a Southern Hemisphere Seagrass Species (Zostera muelleri). *Plant Physiol.* **172**, 272–283 (2016).

70. Ming, R. *et al.* The pineapple genome and the evolution of CAM photosynthesis. *Nat. Genet.* **47**, 1435–1442 (2015).

71. Tang, H., Bowers, J. E., Wang, X. & Paterson, A. H. Angiosperm genome comparisons reveal early polyploidy in the monocot lineage. *Proc. Natl. Acad. Sci. U. S. A.* **107**, 472–477 (2010).

72. Wang, X. *et al.* The genome of the mesopolyploid crop species Brassica rapa. *Nat. Genet.* **43**, 1035–1039 (2011).

73. Kim, Y.-M. *et al.* Genome analysis of Hibiscus syriacus provides insights of polyploidization and indeterminate flowering in woody plants. *DNA Res.* **24**, 71–80 (2017).

74. Hoshino, A. *et al.* Genome sequence and analysis of the Japanese morning glory Ipomoea nil. *Nat. Commun.* **7**, 13295 (2016).

75. Luo, Y.-J. *et al.* The Lingula genome provides insights into brachiopod evolution and the origin of phosphate biomineralization. *Nat. Commun.* **6**, 8301 (2015).

76. Qin, G. *et al.* The pomegranate (Punica granatum L.) genome and the genomics of punicalagin biosynthesis. *Plant J.* **91**, 1108–1128 (2017).

77. Sveinsson, S. *et al.* Phylogenetic pinpointing of a paleopolyploidy event within the flax genus (Linum) using transcriptomics. *Ann. Bot.* **113**, 753–761 (2014).

78. Bombarely, A. *et al.* Insight into the evolution of the Solanaceae from the parental genomes of Petunia hybrida. *Nat Plants* **2**, 16074 (2016).

79. Unver, T. *et al.* Genome of wild olive and the evolution of oil biosynthesis. *Proc. Natl. Acad. Sci. U. S. A.* **114**, E9413–E9422 (2017).

80. Devos, N. *et al.* Analyses of transcriptome sequences reveal multiple ancient large-scale duplication events in the ancestor of Sphagnopsida (Bryophyta). *New Phytol.* **211**, 300–318 (2016).

81. Blanc, G. & Wolfe, K. H. Widespread paleopolyploidy in model plant species inferred from age distributions of duplicate genes. *Plant Cell* **16**, 1667–1678 (2004).

82. Tomato Genome Consortium. The tomato genome sequence provides insights into fleshy fruit evolution. *Nature* **485**, 635–641 (2012).

83. Garsmeur, O. *et al.* Two evolutionarily distinct classes of paleopolyploidy. *Mol. Biol. Evol.* **31**, 448–454 (2014).

84. Freeling, M. The distribution of ancient polyploidies in the plant phylogenetic tree is a spandrel of occasional sex. *Plant Cell* (2017). doi:10.1105/tpc.16.00836

85. Jaillon, O. *et al.* The grapevine genome sequence suggests ancestral hexaploidization in major angiosperm phyla. *Nature* **449**, 463–467 (2007).

86. Tang, H. *et al.* Unraveling ancient hexaploidy through multiply-aligned angiosperm gene maps. *Genome Res.* **18**, 1944–1954 (2008).

87. Clark, J. W. & Donoghue, P. C. J. Constraining the timing of whole genome duplication in plant evolutionary history. *Proc. Biol. Sci.* **284**, (2017).

88. Sayyari, E., Whitfield, J. B. & Mirarab, S. DiscoVista: Interpretable visualizations of gene tree discordance. *Mol. Phylogenet. Evol.* **122**, 110–115 (2018).
