# Supplementary material for: One thousand plant transcriptomes and the phylogenomics of green plants
Source: Nature. 2019 Oct 23;574(7780):679–85. doi: 10.1038/s41586-019-1693-2 (PMC6872490; doi:10.1038/s41586-019-1693-2)
Supplement: Supplementary file 2 — Reporting Summary [file 41586_2019_1693_MOESM2_ESM.pdf]

nature research

Corresponding author(s): James Leebens-Mack & Gane Ka-Shu Wong

Reporting Summary

Nature Research wishes to improve the reproducibility of the work that we publish. This form provides structure for consistency and transparency in reporting. For further information on Nature Research policies, see Authors & Referees and the Editorial Policy Checklist.

Statistical parameters

When statistical analyses are reported, confirm that the following items are present in the relevant location (e.g. figure legend, table legend, main text, or Methods section).

n/a	Confirmed	
☐	☒	The exact sample size (*n*) for each experimental group/condition, given as a discrete number and unit of measurement
☐	☒	An indication of whether measurements were taken from distinct samples or whether the same sample was measured repeatedly
☐	☒	The statistical test(s) used AND whether they are one- or two-sided<br>*Only common tests should be described solely by name; describe more complex techniques in the Methods section.*
☒	☐	A description of all covariates tested
☒	☐	A description of any assumptions or corrections, such as tests of normality and adjustment for multiple comparisons
☐	☒	A full description of the statistics including central tendency (e.g. means) or other basic estimates (e.g. regression coefficient) AND variation (e.g. standard deviation) or associated estimates of uncertainty (e.g. confidence intervals)
☐	☒	For null hypothesis testing, the test statistic (e.g. *F*, *t*, *r*) with confidence intervals, effect sizes, degrees of freedom and *P* value noted<br>*Give P values as exact values whenever suitable.*
☒	☐	For Bayesian analysis, information on the choice of priors and Markov chain Monte Carlo settings
☒	☐	For hierarchical and complex designs, identification of the appropriate level for tests and full reporting of outcomes
☒	☐	Estimates of effect sizes (e.g. Cohen's *d*, Pearson's *r*), indicating how they were calculated
☒	☐	Clearly defined error bars<br>*State explicitly what error bars represent (e.g. SD, SE, CI)*

Our web collection on statistics for biologists may be useful.

Software and code

Policy information about availability of computer code

Data collection	Transcripts were assembled using SOAPdenovo-Trans assembler (version of 2012-04-05); NCBI BLAST, TransRate, CEGMA6 and BUSCO were used to assess assembly quality, translations were performed using TransPipe and Genewise 2.2.2, Gene and species tree
Data analysis	estimates RAxML v. 8.1.17, FastTree-2 v. 2.1.5, and ExaML v. 3.0.14,ASTRAL-II v. 5.0.3 was used to estimate species trees; scripts for post-processing, DiscoVista, of trees - https://github.com/smirarab/1kp ; genome duplications were investigated using the DupPipe, PAML, and the MAPS pipelines including the GuestTreeGen program withinGenPhyloData - https://bitbucket.org/barkerlab/maps ; analysis of gene family expansions included HMMER v3.1b2 and scrips available at https://github.com/GrosseLab/OneKP-gene-family-evo

For manuscripts utilizing custom algorithms or software that are central to the research but not yet described in published literature, software must be made available to editors/reviewers upon request. We strongly encourage code deposition in a community repository (e.g. GitHub). See the Nature Research guidelines for submitting code & software for further information.

Data

Policy information about availability of data

All manuscripts must include a data availability statement. This statement should provide the following information, where applicable:

- Accession codes, unique identifiers, or web links for publicly available datasets
- A list of figures that have associated raw data
- A description of any restrictions on data availability

All data are public: Raw reads in NCBI SRA database - http://www.onekp.com/public_read_data.html ; Assembled transcripts and transcript translations - http://www.onekp.com/public_data.html ; Gene family nucleotide and amino acid fasta files - http://jlmwiki.plantbio.uga.edu/onekp/v2/ ; Multiple sequence alignments, gene trees and species trees for single copy nuclear genes - https://github.com/smirarab/1kp

Field-specific reporting

Please select the best fit for your research. If you are not sure, read the appropriate sections before making your selection.

☐ Life sciences ☐ Behavioural & social sciences ☒ Ecological, evolutionary & environmental sciences

For a reference copy of the document with all sections, see nature.com/authors/policies/ReportingSummary-flat.pdf

Ecological, evolutionary & environmental sciences study design

All studies must disclose on these points even when the disclosure is negative.

Study description	Gene and species phylogenies were estimated in order to infer: relationships across the green tree of life (Viridiplantae), the timing of genome-scale duplication events, and the timing of gene family expansions.
Research sample	RNA was isolated from young vegetative tissue from 1342 samples representing 1147 species across all major subclades of Viridiplantae, glaucophytes (Glaucophyta) and red algae (Rhodophyta) and used to generate RNA seq reads and assemblies.
Sampling strategy	Samples were collected as available in living collections. Species were chosen for RNA seq with a priority to maximize taxonomic diversity across Viridiplantae and outgroups
Data collection	RNA samples were derived from vouchered material in living collections as described in Table 1.
Timing and spatial scale	Samples were collected as available. No attempt was made to control for environmental variation
Data exclusions	RNA samples exhibiting evidence of contamination were excluded from phylogenetic analyses. Contamination was diagnosed through BLAST comparisons to ribosomal RNA and plastid gene databases.
Reproducibility	Bootstrap analyses and Bayesian posterior probabilities were estimated for all nodes in gene trees and species trees.
Randomization	Bootstrap support for nodes gene trees and species trees were estimated in a standard fashion through random resampling of columns in sequence alignments.
Blinding	No blinding was done for any of our analyses.

Did the study involve field work? ☐ Yes ☒ No

Reporting for specific materials, systems and methods

Materials & experimental systems

n/a	Involved in the study
☐	☒ Unique biological materials
☒	☐ Antibodies
☒	☐ Eukaryotic cell lines
☒	☐ Palaeontology
☒	☐ Animals and other organisms
☒	☐ Human research participants

Methods

n/a	Involved in the study
☒	☐ ChIP-seq
☒	☐ Flow cytometry
☒	☐ MRI-based neuroimaging

Unique biological materials

Policy information about availability of materials

Obtaining unique materials	Most samples are available in live collections and/or herbarium vouchers.
